# NudCL2 is required for cytokinesis by stabilizing RCC2 with Hsp90 at the midbody

**DOI:** 10.1093/procel/pwae025

**Published:** 2024-05-27

**Authors:** Xiaoyang Xu, Yuliang Huang, Feng Yang, Xiaoxia Sun, Rijin Lin, Jiaxing Feng, Mingyang Yang, Jiaqi Shao, Xiaoqi Liu, Tianhua Zhou, Shanshan Xie, Yuehong Yang

**Affiliations:** Department of Cell Biology, Institute of Gastroenterology of the Second Affiliated Hospital, Zhejiang University School of Medicine, Hangzhou 310009, China; Department of Cell Biology, Institute of Gastroenterology of the Second Affiliated Hospital, Zhejiang University School of Medicine, Hangzhou 310009, China; Research Center for Children’s Health and Innovation, Binjiang Institute of Zhejiang University, Hangzhou 310053, China; Department of Cell Biology, Institute of Gastroenterology of the Second Affiliated Hospital, Zhejiang University School of Medicine, Hangzhou 310009, China; Department of Cell Biology, Institute of Gastroenterology of the Second Affiliated Hospital, Zhejiang University School of Medicine, Hangzhou 310009, China; Department of Cell Biology, Institute of Gastroenterology of the Second Affiliated Hospital, Zhejiang University School of Medicine, Hangzhou 310009, China; Department of Cell Biology, Institute of Gastroenterology of the Second Affiliated Hospital, Zhejiang University School of Medicine, Hangzhou 310009, China; Department of Cell Biology, Institute of Gastroenterology of the Second Affiliated Hospital, Zhejiang University School of Medicine, Hangzhou 310009, China; Department of Biochemistry, Purdue University, West Lafayette, IN 47907, United States; Department of Cell Biology, Institute of Gastroenterology of the Second Affiliated Hospital, Zhejiang University School of Medicine, Hangzhou 310009, China; Cancer Center, Zhejiang University, Hangzhou 310058, China; Center for RNA Medicine, International Institutes of Medicine, the Fourth Affiliated Hospital of Zhejiang University School of Medicine, Yiwu 322000, China; Children’s Hospital, Zhejiang University School of Medicine, National Clinical Research Center for Child Health, Hangzhou 310052, China; Department of Cell Biology, Institute of Gastroenterology of the Second Affiliated Hospital, Zhejiang University School of Medicine, Hangzhou 310009, China; Cancer Center, Zhejiang University, Hangzhou 310058, China

**Keywords:** cytokinesis, midbody, NudCL2, Hsp90, RCC2

## Abstract

Cytokinesis is required for faithful division of cytoplasmic components and duplicated nuclei into two daughter cells. Midbody, a protein-dense organelle that forms at the intercellular bridge, is indispensable for successful cytokinesis. However, the regulatory mechanism of cytokinesis at the midbody still remains elusive. Here, we unveil a critical role for NudC-like protein 2 (NudCL2), a co-chaperone of heat shock protein 90 (Hsp90), in cytokinesis regulation by stabilizing regulator of chromosome condensation 2 (RCC2) at the midbody in mammalian cells. NudCL2 localizes at the midbody, and its downregulation results in cytokinesis failure, multinucleation, and midbody disorganization. Using iTRAQ-based quantitative proteomic analysis, we find that RCC2 levels are decreased in *NudCL2* knockout (KO) cells. Moreover, Hsp90 forms a complex with NudCL2 to stabilize RCC2, which is essential for cytokinesis. RCC2 depletion mirrors phenotypes observed in NudCL2-downregulated cells. Importantly, ectopic expression of RCC2 rescues the cytokinesis defects induced by NudCL2 deletion, but not *vice versa*. Together, our data reveal the significance of the NudCL2/Hsp90/RCC2 pathway in cytokinesis at the midbody.

## Introduction

Cytokinesis is the final step of cell division, which drives the physical separation of daughter cells (D’Avino et al., 2015). Failure of cytokinesis can result in chromosomal instability and polyploidy, which consequently contributes to the development of pathologies such as cancer (Lens and Medema, 2019). Previous studies characterize cytokinesis as a multistage process governed by precisely temporal and spatial regulation (Fededa and Gerlich, 2012). During anaphase, the protein regulator of cytokinesis 1 (PRC1) initially accumulates at the midzone, promoting microtubule bundling and central spindle formation (Lee et al., 2015). Subsequent recruitment of polo-like kinase 1 (PLK1) to the central spindle activates mitotic kinesin-like protein 2 (MKLP2; also known as KIF20A), aiding in the localization of Aurora B kinase to the central spindle (Gruneberg et al., 2004; Neef et al., 2007). Aurora B then phosphorylates two centralspindlin subunits, mitotic kinesin-like protein 1 (MKLP1; also known as KIF23) and Rac GTPase activating protein 1 (RACGAP1) to promote further central spindle assembly (Douglas et al., 2010; Wagner and Glotzer, 2016). In telophase, the contractile ring progressively compacts the central spindle to form a dense structure at the intercellular bridge called the midbody, which provides a platform necessary for the recruitment and organization of many proteins including citron kinase and Aurora B (Bassi et al., 2013; Capalbo et al., 2019; D’Avino and Capalbo, 2016; Johnson et al., 2017). Finally, abscission is executed by the endosomal sorting complex required for transport (ESCRT) machinery (Mierzwa and Gerlich, 2014). However, knowledge about the molecular mechanism of cytokinesis regulation remains limited.

Regulator of chromosome condensation 2 (RCC2), also known as telophase disc protein of 60 kDa (TD-60), was initially found at the midzone during anaphase and the midbody during cytokinesis (Andreassen et al., 1991; Mollinari et al., 2003). RCC2 localizes to inner centromeres from prophase to metaphase and plays an important role in establishing proper kinetochore-microtubule attachments and chromosome segregation by regulating Aurora B kinase phosphorylation (Papini et al., 2015; Rosasco-Nitcher et al., 2008). Depletion of RCC2 leads to an increase in microtubule density at kinetochores and inter-kinetochore stretch (Papini et al., 2015). However, the precise role of RCC2 in cytokinesis remains uncertain.

NudC-like protein 2 (NudCL2), also known as NudC domain-containing protein 2 (NudCD2), was originally characterized as a homolog of mammalian nuclear distribution gene C (NudC) by our group (Fu et al., 2016; Yang et al., 2010). We find that NudCL2 functions as a cochaperone of heat shock protein 90 (Hsp90) to stabilize several client proteins, including cohesin subunits, lissencephaly protein 1 (LIS1; also known as PAFAH1B1), and myosin-9, which participate in the regulation of chromosome segregation and cell migration (Chen et al., 2020; Yang et al., 2010, 2019). However, the role of NudCL2 in cytokinesis remains unknown.

Here, we provide evidence that the NudCL2/Hsp90/RCC2 pathway is required for cytokinesis at the midbody. We demonstrate that NudCL2, Hsp90, and RCC2 form a biochemical complex and localize at the midbody. NudCL2 plays an important role in cytokinesis by stabilizing RCC2 with Hsp90 at the midbody, providing a previously unexplored mechanism of cytokinesis regulation at the midbody.

## Results

### Downregulation of NudCL2 causes cytokinesis failure

**Figure 1.**
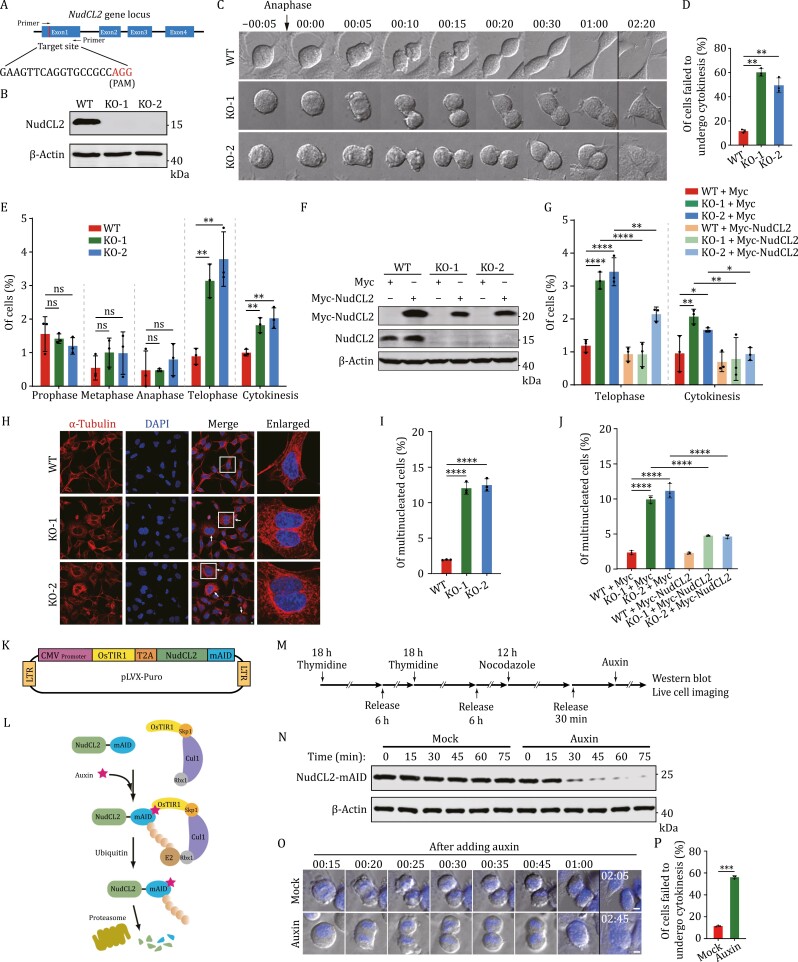
Downregulation of NudCL2 causes cytokinesis failure. (A) Schematic representation of *NudCL2* gene targeting strategy. (B) Western blot analysis of NudCL2 protein in entire WT and *NudCL2* KO HEK-293 cells. (C) Time-lapse DIC images of the live cell imaging experiment of the control or *NudCL2* KO cells. Time point 00:00 (hours:min) refers to the first frame where the separating sister chromatids are observed. (D) Percentage of WT (*n* = 189), KO-1 (*n* = 157) or KO-2 (*n* = 155) cells showing cytokinesis failure was calculated. (E) Cells were stained with DAPI and anti-α-tubulin antibody for immunofluorescence analysis. The percentage of WT (*n* = 905), KO-1 (*n* = 1264), or KO-2 (*n* = 1093) cells in each mitotic phase and cytokinesis was calculated. (F) Cells transfected with Myc or Myc-NudCL2 vector were subjected to Western blot with the antibodies as shown. (G) The percentage of transfected cells in telophase and cytokinesis was calculated using immunofluorescence analysis. The number of calculated cells in WT + Myc, KO-1 + Myc, KO-2 + Myc, WT + Myc-NudCL2, KO-1 + Myc-NudCL2 or KO-2 + Myc-NudCL2 was 1,590, 729, 839, 1,717, 1,101, or 747. One-way ANOVA with multiple comparison corrections was performed. (H) Cells were stained with DAPI and anti-α-tubulin antibody for immunofluorescence. White arrowheads indicate the multinucleated cells. (I) The percentages of the multinucleated cells in WT (*n* = 841), KO-1 (*n* = 1,135), or KO-2 (*n* = 1,009) were calculated. (J) Cells transfected with the indicated vectors were stained to detect DNA and α-tubulin. The percentages of the multinucleated cells (from left to right: *n* = 1,297, 795, 676, 1,429, 952, and 664) were calculated. One-way ANOVA with multiple comparison corrections was performed. (K and L) Schematic illustration of the rapid downregulation strategy of NudCL2 using the AID system. OsTIR1, the Oryza sativa TIR1; T2A, thosea asigna virus 2A; mAID, the degron termed mini-AID. Expressed OsTIR1 binds to the SCF E3 ubiquitin ligase complex (including Skp1, Cul1, and Rbx1) to form a functional complex with the endogenous components in cells. When Auxin interacts with OsTIR1 and mAID, SCF-OsTIR1 recruits an E2 ligase to induce polyubiquitylation and degradation of NudCL2-mAID protein. (M) The workflow of NudCL2 downregulation by AID system in cells stably expressing OsTIR1 and NudCL2-mAID using *NudCL2* KO cell (NudCL2-mAID cells). (N) The NudCL2-mAID cells were synchronized into anaphase by thymidine-nocodazole block and release procedure, and treated with or without auxin. Then the entire cells were harvested immediately at different time points, and subjected to Western blot with anti-mAID antibody. (O and P) The synchronized NudCL2-mAID cells were subjected to live cell imaging experiments. The first frame of time-lapse DIC images was taken at 15 min after auxin and Hoechst 33342 addition and the time point was marked as 00:15 (hours:min). Percentage of mock (*n* = 131) or auxin-treated (*n* = 112) cells showing cytokinesis failure was calculated. β-Actin, a loading control. Scale bars, 5 μm. Higher magnifications of the boxed regions are displayed. Quantitative data are expressed as the mean ± SD (from three biological replicates). The *P* values were calculated using Student’s *t*-test. *P < 0.05, **P < 0.01, ***P < 0.001, ****P < 0.0001; ns, no signiﬁcance.

To explore the role of NudCL2 in cytokinesis, we employed CRISPR/Cas9-mediated genome editing to knockout (KO) *NudCL2* in HEK-293 cells (Fig. 1A). Sanger sequencing revealed indels causing frameshift mutations at the *NudCL2* DNA locus (Fig. S1A). Immunoblotting confirmed the absence of NudCL2 protein in KO cell lines (KO-1 and KO-2) (Fig. 1B). Live cell imaging showed that cells lacking NudCL2 failed to undergo cytokinesis, and approximately 50% of *NudCL2* KO cells exhibited full-cleavage furrow ingression but eventually underwent cleavage furrow regression (Figs. 1C, 1D, S1B, S1C and Movies S1–6). In addition, immunofluorescence analysis revealed a higher proportion of *NudCL2* KO cells in telophase and cytokinesis compared to wild-type (WT) cells, and ectopic expression of Myc-NudCL2 in *NudCL2* KO cells efficiently reversed these phenotypes (Fig. 1E–G). These data suggest that deletion of NudCL2 causes cytokinesis failure. Given the crucial role of cytokinesis in proper chromosome segregation and cell division, failure of which leads to multinucleation (Krajcovic et al., 2011; Rengstl et al., 2013; Xu et al., 2019), we further conducted immunofluorescence analysis to validate the involvement of NudCL2 in cytokinesis. Our results demonstrated a significant increase in multinucleation upon NudCL2 deletion compared to the control, which was effectively reversed by ectopic expression of NudCL2 (Fig. 1H–J). A similar phenotype was observed in *NudCL2* KO HeLa cells (Fig. S2A–D). These data indicate that NudCL2 is required for cytokinesis.

To directly assess the role of NudCL2 in cytokinesis, we employed the auxin-inducible degron (AID) system to induce acute downregulation of NudCL2 protein level after anaphase, then performed live cell imaging experiment to observe the cytokinesis phenotype (Natsume et al., 2016; Nishimura et al., 2009). The results displayed that rapid depletion of NudCL2 during cytokinesis resulted in regression of cleavage furrow and consequently led to cytokinesis failure (Fig. 1K–P, Movies S7 and S8). These results suggest a direct function of NudCL2 in cytokinesis.

### Loss of NudCL2 disrupts the midbody organization

**Figure 2.**
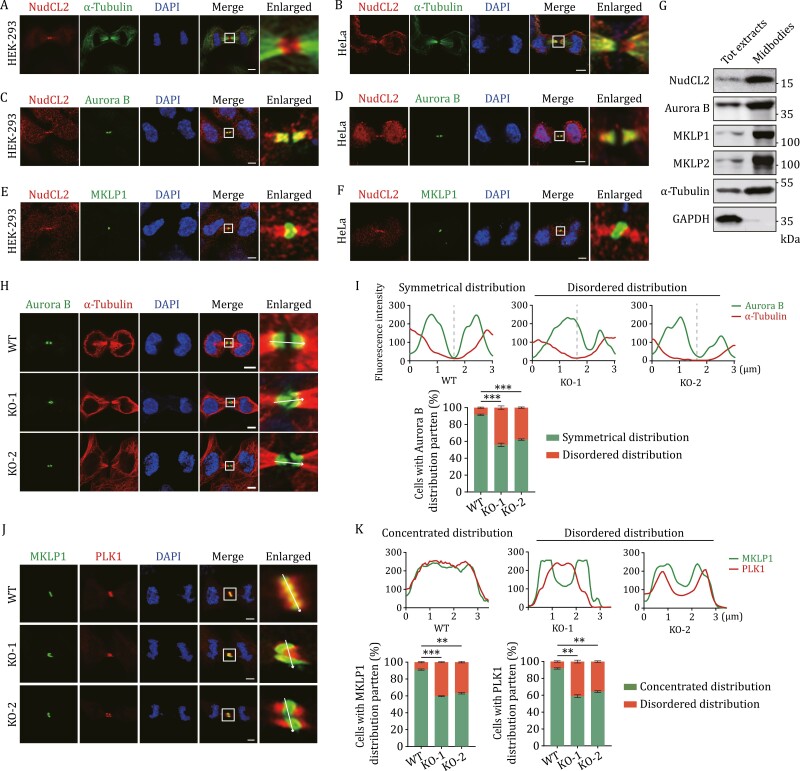
Loss of NudCL2 disrupts the midbody organization. (A–F) Cells were fixed and subjected to immunofluorescence analyses with the antibodies as shown. (G) HEK-293 cells were synchronized with a thymidine-nocodazole block, released, and harvested. The proteins from the total protein extracts (Tot extracts) and midbodies were extracted and subjected to Western blot analysis with the indicated antibodies. (H) Control and *NudCL2* KO cells were fixed and subjected to immunofluorescence analysis with anti-Aurora B and anti-α-tubulin antibodies. (I) Intensity line scan plots for corresponding proteins of images. The frequencies of WT (*n* = 166), KO-1 (*n* = 168), or KO-2 (*n* = 166) cells with mislocalization of Aurora B at the midbody were calculated. (J) Control and *NudCL2* KO cells were fixed and subjected to immunofluorescence analysis with anti-MKLP1 and anti-PLK1 antibodies. (K) Intensity line scan plots for corresponding proteins of images. The frequencies of WT (*n* = 158), KO-1 (*n* = 158), or KO-2 (*n* = 157) cells with mislocalization of MKLP1 (K) or PLK1 (L) at the midbody were calculated. DNA was visualized with DAPI. Scale bars, 5 μm. Higher magniﬁcations of the boxed regions are displayed. Intensity of line scan plots was quantified and graphed using ImageJ software. White arrows indicate the direction of line scan plots. Quantitative data are expressed as the mean ± SD (from three biological replicates). The *P* values were calculated using Student’s *t*-test. **P < 0.01, ***P < 0.001; ns, no significance.

To further address the function of NudCL2 in cytokinesis, we detected the localization of NudCL2 during this process and found that NudCL2 accumulated at the midbody in both HEK-293 and HeLa cells (Fig. 2A and 2B). Further immunofluorescence staining showed that NudCL2 predominantly co-localizes with Aurora B rather than MKLP1, implying that it was mainly distributed at the midbody arm region (Fig. 2C–F). Moreover, we purified the midbodies from synchronized telophase HEK-293 cells and carried out Western blot experiments. Our results revealed that NudCL2 co-purified with midbody proteins, including Aurora B, MKLP1, and MKLP2 (Fig. 2G). Thus, our data indicate that NudCL2 is a midbody-associated protein.

The midbody serves as a key platform for recruiting and organizing proteins that regulate cytokinesis, and mislocalization of these proteins can lead to cytokinesis failure (Capalbo et al., 2019; D’Avino and Capalbo, 2016). Since NudCL2 localizes at the midbody and its deletion results in cytokinesis failure, we investigated whether loss of NudCL2 may disrupt midbody organization. Immunofluorescence experiments were performed to detect the localization of three characterized midbody components: Aurora B, MKLP1, and PLK1 (a marker of the midbody core). Our results showed that Aurora B exhibited symmetrically distributed at the midbody arm in control cells, while it lost the precise arrangement and exhibited a dispersed distribution pattern in *NudCL2* KO cells (Figs. 2H, 2I and S3A). Meanwhile, MKLP1 and PLK1 precisely gathered at the center of the midbody core and exhibited a concentrated distribution pattern in control cells, but this pattern was disordered in *NudCL2* KO cells (Figs. 2J, 2K and S3B). Similar results were found in *NudCL2*-deleted HeLa cells (Fig. S4A–C). Together, our data imply that the deletion of NudCL2 disrupts the midbody organization.

### Loss of NudCL2 decreases the protein level of RCC2 in the midbody

**Figure 3.**
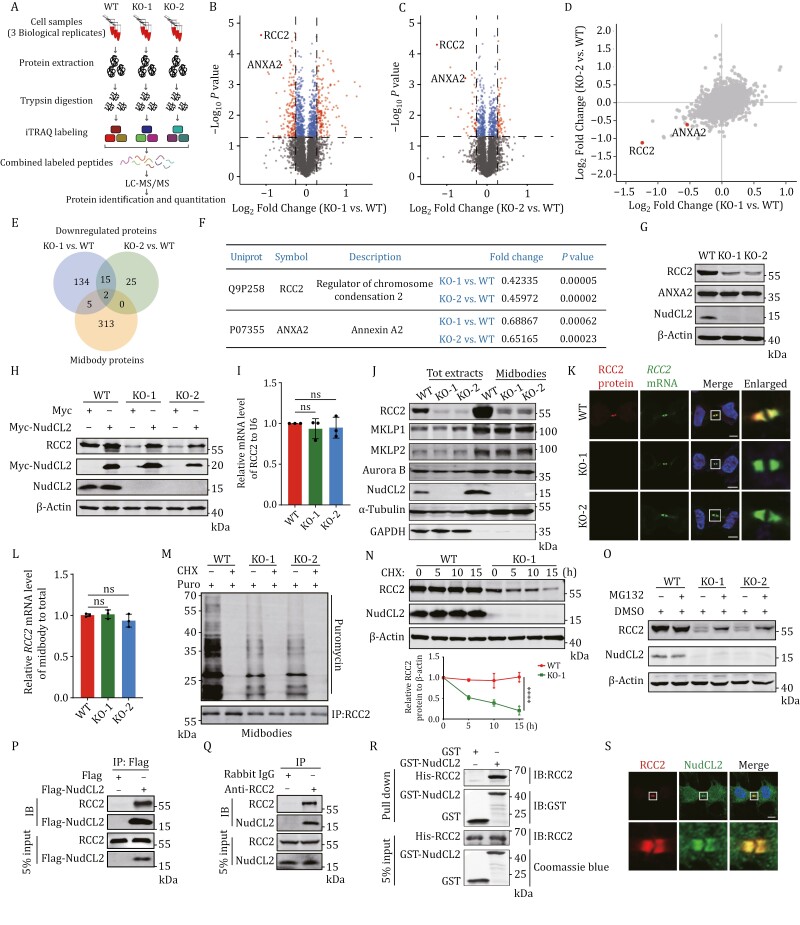
Loss of NudCL2 decreases the RCC2 protein levels in the midbody. (A) Schematic representation of the isobaric tags for relative and absolute quantitation (iTRAQ)-based quantitative proteomic analysis. (B and C) Volcano plot showing *P* values (−log_10_) versus the protein ratio of KO-1/WT cells (log_2_) or KO-2/WT cells (log_2_). Proteins exhibiting a fold change > 1.2 or < 0.83, *P* < 0.05 were defined as “differentially expressed proteins” (red dots). Others were defined as “no change” (blue dots). (D) Scatter plot showing the protein ratio of KO-1/WT cells (log_2_) versus KO-2/WT cells (log_2_). Each point represents a single protein identiﬁed. (E) Venn diagram showing the overlap among the proteins downregulated in KO-1 and KO-2 of the quantitative proteomic analysis and the midbody proteins from the MiCroKiTS database. (F) Two midbody proteins that overlap in Fig. 3E are shown. (G) Western blot analysis of proteins from entire control and *NudCL2* KO cells using the indicated antibodies. (H) Western blot analysis of proteins from entire control and *NudCL2* KO cells transfected with Myc or Myc-NudCL2 vector with the indicated antibodies. (I) The qRT-PCR analysis of *RCC2* mRNA in control and *NudCL2* KO cells. ns, no significance, Student’s *t*-test. (J) Western blot analysis of total protein extracts (Tot extracts) and midbodies purified from control and *NudCL2* KO cells with the indicated antibodies. (K) Control and *NudCL2* KO cells were fixed and subjected to smFISH to detect *RCC2* mRNA, followed by immunofluorescence analysis with the anti-RCC2 antibody. (L) The qRT-PCR analysis of *RCC2* mRNA in total extracts and midbodies purified from synchronized telophase control and *NudCL2* KO HEK-293 cells. ns, no significance, Student’s *t*-test. (M) Cells were synchronized with a thymidine-nocodazole block/release and labeled with puromycin with or without CHX. Then, the midbodies were purified and subjected to immunoprecipitation analysis with anti-RCC2 antibody. (N) Western blot analysis of proteins from entire control and *NudCL2* KO cells treated with 100 μg/mL CHX using the indicated antibodies. The intensity of each band was measured using ImageJ software. The relative amounts of RCC2 were calculated after normalization (RCC2/β-actin). ****P < 0.0001, two-way ANOVA. (O) Western blot analysis of protein from entire cells treated with 1 μmol/L MG132 for 24 h. (P) The entire cells were transfected with the indicated vectors and immunoprecipitation analysis was performed using anti-Flag antibody. (Q) Immunoprecipitation analysis was performed using the indicated antibodies. (R) *In vitro* GST pull-down assays using purified GST, GST-NudCL2, and His-RCC2 protein. (S) Cells were subjected to immunofluorescence analyses with the indicated antibodies. DNA was visualized with DAPI. Higher magniﬁcations of the boxed regions are displayed below. Scale bars, 5 μm. β-Actin, a loading control. 5% of the total input is shown.

Since NudCL2 localizes at the midbody and is required for cytokinesis, we investigated the potential regulator involved in NudCL2-mediated cytokinesis regulation by isobaric tags for relative and absolute quantitation (iTRAQ)-based quantitative proteomic analysis. Our analysis revealed hundreds of differentially expressed proteins (KO/WT fold change > 1.2 or < 0.83, *P* < 0.05) in *NudCL2* KO cells (Fig. 3A–D, Dataset S1). Then, we compared downregulated proteins in *NudCL2* KO-1 and KO-2 cells with midbody proteins from MiCroKiTS 4.0 database (Huang et al., 2015) (Dataset S2) and found two midbody proteins, regulator of chromosome condensation 2 (RCC2) and annexin A2 (ANXA2), that overlapped in these datasets (Fig. 3E and 3F). To validate the regulation of NudCL2 on the protein levels of RCC2 and ANXA2, we performed Western blot analysis and observed a substantial decrease only in RCC2 protein levels following NudCL2 downregulation (Figs. 3G and S5), which was rescued by ectopic expression of NudCL2 (Fig. 3H). Similar results were observed in *NudCL2* KO HeLa cells (Fig. S6A and S6B).

Quantitative real-time polymerase chain reaction (qRT-PCR) experiments showed no significant change in *RCC2* mRNA levels between *NudCL2* KO and control cells (Fig. 3I). However, we observed a noticeable reduction in both total RCC2 protein levels and RCC2 localization at the midbody upon NudCL2 deletion (Fig. 3J and 3K). Interestingly, *RCC2* mRNA was also detected at the midbody by single-molecule fluorescent *in situ* hybridization (smFISH), which is shown to co-localize with RCC2 protein (Figs. 3K and S7A). The *RCC2* mRNA levels in the midbody remained unaffected by NudCL2 deletion (Fig. 3L). These data suggest a specific role of NudCL2 in the regulation of RCC2 protein at the midbody. Furthermore, our observations revealed the presence of newly synthesized RCC2 peptides at the midbody (Figs. 3M, S7B and S7C). These peptides exhibited a reduction upon NudCL2 deletion, suggesting NudCL2’s involvement in regulating the nascent peptides of RCC2 at the midbody.

To further examine whether NudCL2 regulates the stability of RCC2, we treated cells with cycloheximide (CHX) and observed accelerated degradation of RCC2 protein in *NudCL2* KO cells compared to controls (Figs. 3N and S6C). The degradation of RCC2 protein induced by NudCL2 KO was rescued by the proteasome inhibitor MG132, accompanied by increased ubiquitination of RCC2 (Figs. 3O, S8A and S8B). Meanwhile, the lysosome inhibitors ammonium chloride (NH4Cl) or bafilomycin A1 (BafA1) failed to reverse the degradation of RCC2 protein induced by NudCL2 KO (Fig. S8C and S8D), confirming that RCC2 protein is mainly degraded by the ubiquitin-dependent proteasome pathway in *NudCL2* KO cells. Additionally, co-immunoprecipitation (Co-IP) and GST-pull-down results displayed that NudCL2 interacted with RCC2 *in vivo* and *in vitro* (Fig. 3P–R). Moreover, co-localization of NudCL2 and RCC2 was observed at the midbody during cytokinesis (Figs. 3S and S9). These data suggest that NudCL2 plays a crucial role in stabilizing RCC2 protein in the midbody.

### Hsp90 interacts with NudCL2 and RCC2 and regulates RCC2 stability

**Figure 4.**
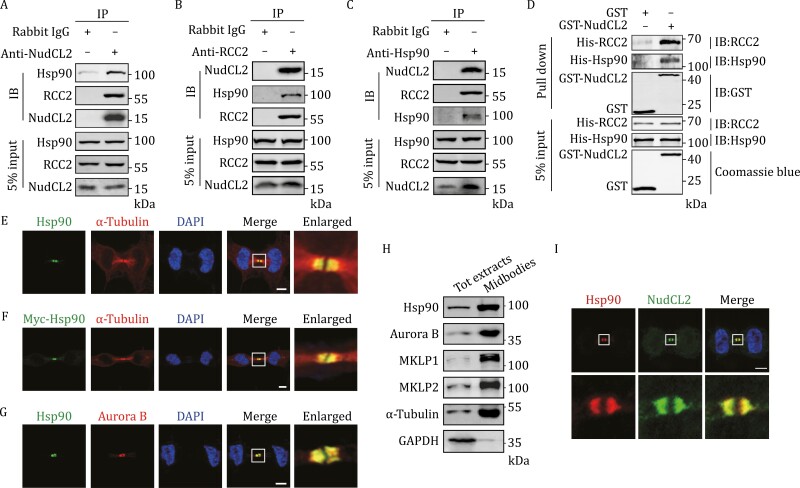
RCC2 interacts with NudCL2 and Hsp90. (A–C) Immunoprecipitation analysis was performed using the indicated antibodies. (D) *In vitro* GST pull-down assays using purified GST-NudCL2 with His-RCC2 and His-Hsp90 proteins. (E) Cells were subjected to immunofluorescence analysis with the indicated antibodies. (F) Cells transfected with Myc-Hsp90 vector were subjected to immunofluorescence analysis with the indicated antibodies. (G) Cells were subjected to immunofluorescence analyses with the indicated antibodies. (H) Western blot analysis of total protein extracts (Tot extracts) and midbodies purified from telophase cells with the indicated antibodies. (I) Cells were subjected to immunofluorescence analyses with the indicated antibodies. DNA was visualized with DAPI. Scale bars, 5 μm. Higher magniﬁcations of the boxed regions are shown. 5% of the total input is shown.

Our previous data had shown that NudCL2 may act as Hsp90 cochaperone to regulate protein stability (Chen et al., 2020; Yang et al., 2010, 2019), which prompted us to detect whether Hsp90 is involved in the regulation of RCC2 stabilization. We first detected the interaction among Hsp90, NudCL2, and RCC2. Immunoprecipitation assays showed that Hsp90 interacted with NudCL2 and RCC2 *in vivo* (Fig. 4A–C). GST-pull-down experiments confirmed the interaction of Hsp90, NudCL2, and RCC2 *in vitro* (Fig. 4D). Considering that the NudC family comprises NudC and NudCL in addition to NudCL2 (Fu et al., 2016; Taipale et al., 2014), we detected the interaction of these proteins with Hsp90 and RCC2. The results showed that RCC2 interacted with NudCL2 and Hsp90, but not with NudC and NudCL *in vivo* (Fig. S10A–C), suggesting that NudCL2 specifically interacts with Hsp90 and RCC2.

**Figure 5.**
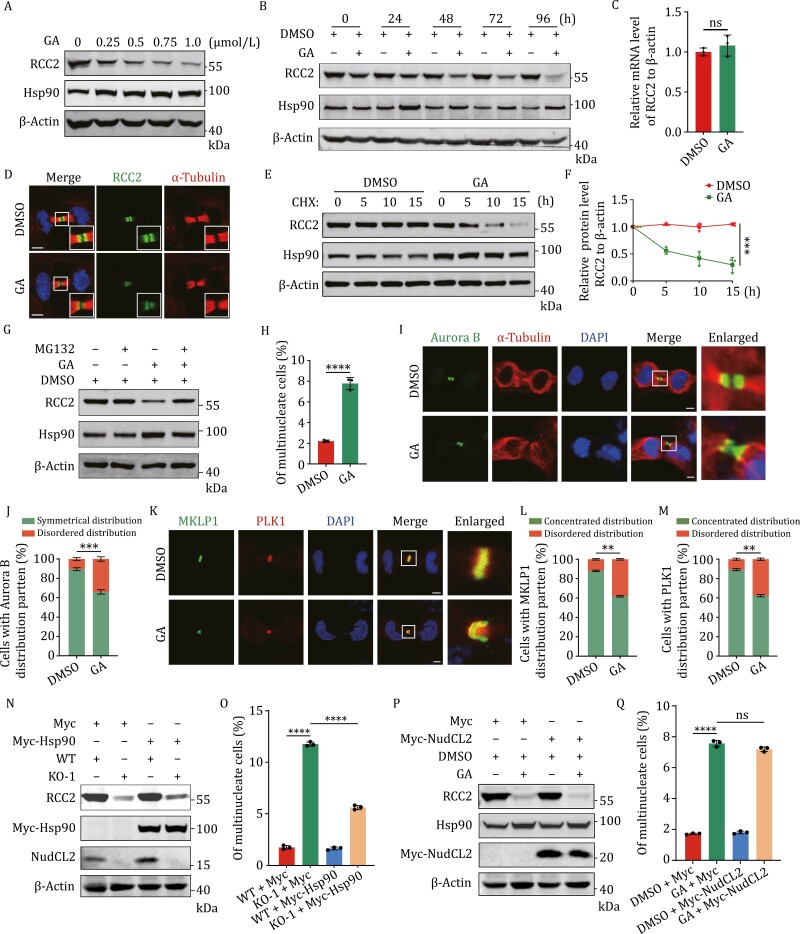
Hsp90 is involved in cytokinesis by regulating RCC2 with NudCL2. (A and B) The entire cells treated with different concentrations of GA for 48 h (A) or 0.5 μmol/L GA at the indicated time points (B) were subjected to Western blot analyses with the indicated antibodies. (C) The entire cells were treated with 0.5 μmol/L GA for 48 h and subjected to qRT-PCR analysis. (D and E) Cells were treated with 0.5 μmol/L GA. After 24 h, cells were added with 100 μg/mL CHX and harvested at the indicated times, then subjected to Western blot analysis using the indicated antibodies. (D) Cells were subjected to immunofluorescence analysis with the indicated antibodies. (E and F) The entire cells were treated with 0.5 μmol/L GA. After 24 h, cells were added with 100 μg/mL CHX and harvested at the indicated times, then subjected to Western blot analysis using the indicated antibodies (E). The intensity of each band was measured using ImageJ software and the relative protein levels of RCC2 were normalized to β-actin (F). Quantitative data are shown from three biological replicates. All values are expressed as mean ± SEM; statistical analyses were performed using two-way ANOVA. (G) The entire cells were treated with 0.5 μmol/L GA for 24 h, then incubated with 1 μmol/L MG132 for another 24 h and subjected to Western blot analysis. β-Actin, a loading control. (H) Cells were stained with DAPI and anti-α-tubulin antibody. The percentages of the multinucleation in DMSO (*n* = 2,218) and GA-treated (*n* = 1,469) cells were calculated. (I–M) Cells were subjected to an immunofluorescence experiment as described in Fig. 2H–K. The frequencies of DMSO or GA-treated cells with Aurora B (*n* = 292 or 206), MKLP (*n* = 209 or 220) or PLK1 (*n* = 148 or 162) mislocalization were calculated. (N and O) Cells transfected with Myc or Myc-Hsp90 were subjected to Western blot and immunofluorescence analyses as described in Fig. 1H. The percentages of the multinucleation (from left to right: *n* = 1,662, 1,736, 1,102, and 1,797) were calculated. (P and Q) Cells treated with or without GA with the indicated vectors were subjected to Western blot and immunofluorescence analyses as described in Fig. 1H. The percentages of the multinucleation (from left to right: *n* = 3,785, 2,912, 4,937, and 4,195) were calculated. DNA was visualized with DAPI. Scale bars, 5 μm. Higher magnifications of the boxed regions are shown. β-Actin, a loading control. Quantitative data are expressed as the mean ± SD from the three biological replicates. The *P* values were calculated using Student’s *t*-test. ***P < 0.001, ****P < 0.0001; ns, no significance.

Moreover, the immunofluorescence data showed that Hsp90 accumulated at the midbody (Figs. 4E–H and S11) and co-localized with NudCL2 during cytokinesis (Fig. 4I). To further detect whether Hsp90 is involved in the RCC2 stability as NudCL2, we treated cells with the Hsp90 inhibitors geldanamycin (GA) or radicicol (RA) and observed a significant reduction in RCC2 protein levels without affecting its mRNA expression (Figs. 5A–C, S12A and S12B). Additionally, RCC2 signals at the midbody decreased following Hsp90 inhibition (Figs. 5D and S12C). CHX chase analysis revealed accelerated degradation of RCC2 protein upon Hsp90 inhibition (Fig. 5E and 5F), and the decreased RCC2 protein induced by Hsp90 inhibition could be reversed by MG132 treatment (Fig. 5G). Together, these data suggest that Hsp90 is required for RCC2 stabilization by interacting with NudCL2 and RCC2.

### Hsp90 is involved in cytokinesis by regulating RCC2 with NudCL2

Since NudCL2 is required for cytokinesis, Hsp90 interacts with NudCL2 to stabilize RCC2 and localizes to the midbody, we further examined whether Hsp90 has a role in cytokinesis. Our data showed inhibition of Hsp90 ATPase activity caused a significant increase in multinucleation compared to the control (Fig. 5H). Meanwhile, Hsp90 inhibition also led to the mislocalization of midbody proteins Aurora B, MKLP1, and PLK1 (Fig. 5I–M). Aurora B was observed to spread from the midbody arm into the midbody core (Fig. 5I), while MKLP1 and PLK1 failed to precisely gather at the center of the midbody core in GA-treated cells (Fig. 5K), implying that Hsp90 inhibition disrupts the midbody organization.

Given that depletion of NudCL2 or inhibition of Hsp90 ATPase activity destabilizes RCC2 and causes cytokinesis defects, we hypothesized that Hsp90 might contribute to cytokinesis by regulating RCC2 along with NudCL2. To test this hypothesis, we carried out rescue experiments and found that exogenous expression of Hsp90 efficiently restored the protein level of RCC2 and reversed the functional defect induced by NudCL2 deletion, whereas ectopic expression of NudCL2 failed to rescue the decrease of RCC2 protein and the cytokinesis defect caused by Hsp90 inhibition (Fig. 5N–Q). These data suggest that Hsp90 plays a role in cytokinesis by regulating RCC2 in conjunction with NudCL2.

### RCC2 participates in cytokinesis as the downstream target of NudCL2.

**Figure 6.**
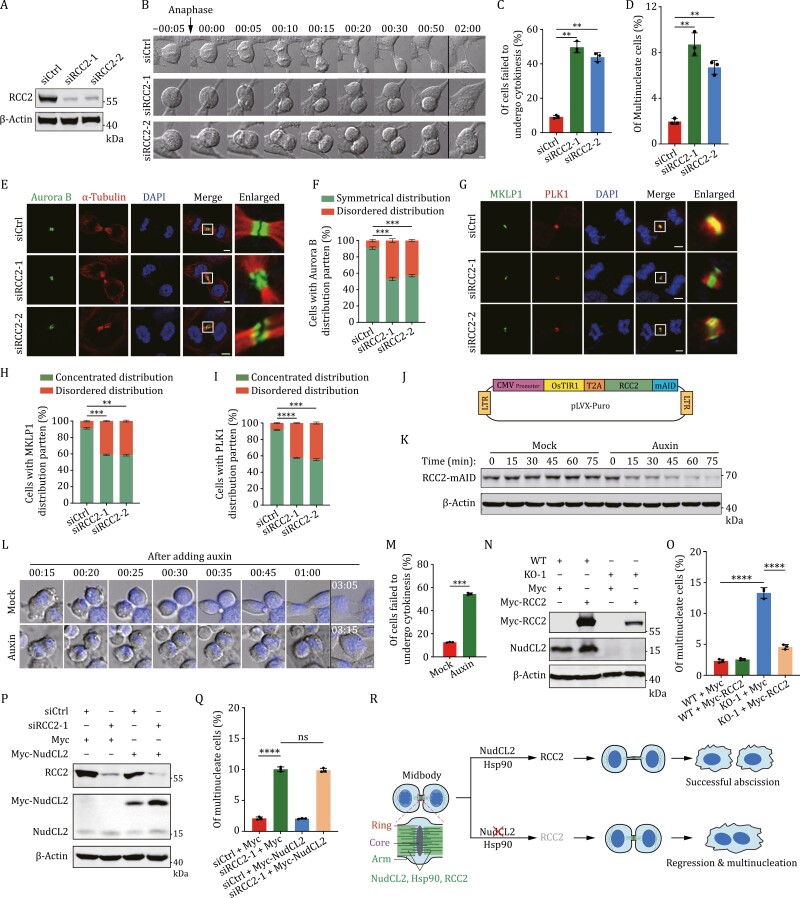
RCC2 participates in cytokinesis as the downstream target of NudCL2. (A) HEK-293 cells transfected with control or RCC2 siRNAs were subjected to Western blot analysis from the entire cells. (B and C) Time-lapse DIC images of the live cell imaging experiment of cells transfected with RCC2 siRNAs. Percentage of siCtrl (*n* = 186), siRCC2-1 (*n* = 101) or siRCC2-2 (*n* = 75) cells showing cytokinesis failure was calculated. (D) The transfected cells with the siRNAs as shown were fixed and stained to detect DNA and α-tubulin. The percentages of the multinucleation in siCtrl (*n* = 1,069), siRCC2-1 (*n* = 1,117) and siRCC2-2 (*n* = 1,135) cells were calculated. (E–I) HEK-293 cells transfected with the indicated siRNAs were fixed and subjected to immunofluorescence analyses with anti-α-tubulin and anti-Aurora B (E), anti-MKLP1, and anti-PLK1 (G) antibodies. The frequencies of siCtrl, siRCC2-1 or siRCC2-2 cells with Aurora B (*n* = 161, 162, or 161), MKLP1 (*n* = 160, 161, or 159) or PLK1 (*n* = 157, 158, or 157) mislocalization were calculated. (J) Schematic illustration of the rapid downregulation strategy of RCC2 using the AID system. (K) The *RCC2* KO cells expressing OsTIR1-T2A-RCC2-mAID were synchronized into anaphase by thymidine-nocodazole blocking and releasing for 30 min. Then cells were treated with or without auxin and harvested immediately at different time points, and subjected to Western blot with anti-mAID antibodies from the entire cells. (L and M) Time-lapse DIC images of the live cell imaging experiment of the *RCC2* KO cells expressing OsTIR1-T2A-RCC2-mAID. Cells were synchronized into anaphase by thymidine-nocodazole blocking and  releasing  for 30 min, then treated with auxin and Hoechst 33342, after 15 min, subjected to the live cell imaging experiment. The first frame of images was taken at 15 min after auxin addition and the time point was marked as 00:15 (hours:min). Percentage of mock (*n* = 142) or auxin-treated (*n* = 134) cells showing cytokinesis failure was calculated. (N and O) Control and *NudCL2* KO cells were transfected with Myc or Myc-RCC2 vector and subjected to Western blot from the entire cells and immunofluorescence analyses using the indicated antibodies. The percentages of the multinucleated cells (from left to right: *n* = 770, 923, 708, and 566) were calculated. (P and Q) Control or RCC2 siRNA cells transfected with Myc or Myc-NudCL2 vector were subjected to Western blot from the entire cells and immunofluorescence analyses using the indicated antibodies. The percentages of the multinucleated cells (from left to right: *n* = 2,007, 3,011, 1,505, and 2,403) were calculated. (R) Working model for the role of NudCL2 in cytokinesis. NudCL2, Hsp90, the mRNA, and the protein of RCC2 all localize at the midbody arm. NudCL2 collaborates with Hsp90 to stabilize RCC2 protein and ensure successful abscission, while loss of NudCL2 decreases RCC2 at the midbody to result in cleavage furrow regression and multinucleation. β-Actin, a loading control. Scale bars, 5 μm. Quantitative data are expressed as the mean ± SD (from three biological replicates). The *P* values were calculated using Student’s *t*-test. **P < 0.01, ***P < 0.001, ****P < 0.0001; ns, no significance.

Given RCC2’s localization at the midbody during cytokinesis and its stabilization by NudCL2, we investigated whether RCC2 is involved in cytokinesis. We employed two small interference RNAs (siRNA) targeting two *RCC2* mRNA regions (siRCC2-1 and -2). Western blot results showed that RCC2 protein levels were obviously decreased after transfection with two RCC2-specific siRNAs (Fig. 6A). Importantly, the live cell imaging experiments displayed that depletion of RCC2 led to a defect in cytokinesis, with approximately 50% of RCC2-depleted cells exhibited full-cleavage furrow ingression but then underwent cleavage furrow regression (Fig. 6B, 6C and Movies S9–11). In addition, RCC2-depletion resulted in multinucleation and midbody disorganization (Fig. 6D–I). To further verify the role of RCC2 in cytokinesis, acute downregulation of RCC2 protein was induced by the AID system after anaphase. Time-lapse imaging data displayed that lacking RCC2 led to the furrow regression similar to that of NudCL2 downregulation (Fig. 6J–M, Movies S12 and S13). Together, our data suggest that RCC2 plays an important role in cytokinesis.

Since NudCL2 interacts with and stabilizes RCC2, and either *NudCL2* deletion or RCC2 depletion may induce failure of cytokinesis, we predicted that RCC2 may be involved in NudCL2-mediated cytokinesis regulation. To test this hypothesis, we conducted rescue experiments. Our data showed that Myc-RCC2 was able to localize at the midbody in *NudCL2* KO cells, and ectopic expression of RCC2 effectively rescued multinucleation caused by NudCL2 deletion (Figs. 6N, 6O and S13). In contrast, ectopic expression of NudCL2 failed to rescue this phenotype induced by RCC2 depletion (Fig. 6P and 6Q). Collectively, our data suggest that RCC2 regulates cytokinesis by acting as a downstream target of NudCL2.

## Discussion

Our previous studies have shown that NudCL2 is an Hsp90 cochaperone and plays important roles in chromosome segregation and cell migration by regulating the stability of its client proteins (Chen et al., 2020; Yang et al., 2010, 2019). However, the role of NudCL2 during cytokinesis is not clear. Here, we observe the localization of NudCL2 and Hsp90 at the midbody during cytokinesis, shedding light on another scenario where NudCL2 collaborates with Hsp90 to oversee cytokinesis regulation. NudCL2, Hsp90, and RCC2 form a complex to localize at the midbody, which is critical for ensuring successful cytokinesis (Fig. 6R).

We have previously found that suppression of NudCL2 caused chromosome misalignment and premature sister chromatid separation (Yang et al., 2019), and other studies also showed that cytokinesis phenotypes are frequently revealed when chromatin is abnormally segregated (Lange et al., 2002; Mendoza and Barral, 2008; Terada, 2001; Wang et al., 2008). The protein rapid depletion using auxin-inducible degron (AID) technology is helpful to study protein direct function and observe an immediate phenotype in living cells (Natsume et al., 2016; Nishimura et al., 2009). To further verify the direct role of NudCL2 in cytokinesis, we employ the AID system to selectively reduce NudCL2 protein levels after anaphase and find that rapid downregulation of NudCL2 in synchronized NudCL2-mAID cells causes cytokinesis failure, suggesting the direct role of NudCL2 in cytokinesis. Further in-depth investigation is clearly needed to explore the potential connection between previous mitotic defects and cytokinesis abnormalities.

Accumulating studies have indicated that RCC2 plays essential roles in a number of cellular processes including cell cycle progression, cell migration, proliferation, and invasion (Calderon-Aparicio and Bode, 2021; Guo et al., 2020; Papini et al., 2015; Song et al., 2018). The localization of RCC2 at the midbody during cytokinesis is well-documented (Andreassen et al., 1991; Mollinari et al., 2003). However, there is limited knowledge regarding the involvement of RCC2 in cytokinesis. Here, our data show that RCC2 plays an important role in cytokinesis, and the NudCL2/Hsp90 complex is involved in the regulation of RCC2 stability to participate in cytokinesis. Given that RCC2 is reported to be crucial for centromeric Aurora B autophosphorylation during prometaphase (Papini et al., 2015; Rosasco-Nitcher et al., 2008), and the activity of Aurora B at the midbody is required for cytokinesis (Broad and DeLuca, 2020), it would be intriguing to investigate whether RCC2 influences Aurora B activity at the midbody during cytokinesis.

Although it has been reported that RCC2 could be regulated by bromodomain-containing protein 4 (BRD4) and insulin-like growth factor 2 mRNA binding protein 3 (IGF2BP3) at the transcription level (Wu et al., 2022; Zhang et al., 2022), the regulation of RCC2 protein stability remains little known. Here, we find that the RCC2 protein levels were decreased after NudCL2 knockout based on iTRAQ-based proteomic analysis. NudCL2, Hsp90, and RCC2 could form a biochemical complex to modulate the RCC2 protein stability and co-localize at the midbody. Furthermore, our previous study suggests that NudCL2 promotes the chaperone function of Hsp90 by modulating the ATPase activity of Hsp90 (Yang et al., 2019), combined with our result that either NudCL2 deletion or Hsp90 inhibition decreases the RCC2 protein stability, we speculate that Hsp90’s ATPase activity may contribute to the formation of the NudCL2/Hsp90/RCC2 complex to stabilize the RCC2 protein.

Current studies reveal that mRNAs can be transported and localized to the special organelles, including dendrites, axons, cilia, and centrosomes, to control *in situ* protein synthesis and local cell physiology (Besse and Ephrussi, 2008; Das et al., 2021). Current evidence suggest that the midbody is enriched in mRNAs and is the site of ribonucleoprotein assembly (Capalbo et al., 2019; Farmer et al., 2023; Park et al., 2023; Skop et al., 2004). Here, our data show that *RCC2* mRNA accumulates at the midbody and co-localizes with its protein, and the nascent polypeptides of RCC2 are detected in the midbody, suggesting that the local translation of RCC2 might occur at the midbody. Meanwhile, deletion of NudCL2 decreases the nascent peptides of RCC2 but not its mRNA level at the midbody, suggesting the potential role of NudCL2 in the regulation of local translation of RCC2. However, further experimental work is required to elucidate the regulatory mechanism of the local translation of RCC2.

In conclusion, our study provides evidence that the NudCL2/Hsp90/RCC2 pathway is required for cytokinesis at the midbody in mammalian cells, which enhances our comprehension of the biological functions of the NudCL2 protein and provides fresh insights into the molecular mechanisms underlying cytokinesis. Given that multinucleation generated through cytokinesis defects may further increase the probability of aneuploidy, which contributes to genetic diversification within many diseases, especially in tumors (Lens and Medema, 2019). In the future, it is worth exploring the role of NudCL2/Hsp90/RCC2 pathway in chromosomal instability and diseases associated with cytokinesis defects.

## Materials and methods

### Plasmids and small interfering RNAs (siRNAs)

The human *Myc-NudCL2*, *GST-NudCL2*, *Myc-Hsp90*, and *His-Hsp90* vectors were previously constructed by our group (Chen et al., 2020; Li et al., 2019; [Bibr CIT0053][Bibr CIT0052]). Full-length human *RCC2* cloned by PCR from *pSA-RCC2* (Song et al., 2018) (a kind gift from Yuxin Yin, Peking University Institute of Systems Biomedicine, Beijing, China) was inserted into pcDNA 3.1/Myc-His (Myc/His-tag vector, Invitrogen, USA) and pET-28a (His-tag vector, Novagen, China). The Oryza sativa TIR1 (OsTIR1) F-box, thosea asigna virus 2A (T2A), and the degron termed mini-AID (mAID) were cloned by PCR from pEF-osTIR1-T2A-mCherry-AID vector (Addgene, No. 160042). OsTIR1, T2A, NudCL2/RCC2, and mAID were inserted into the pLVX-Puro vector (Clontech, No. 632164). RCC2-∆N, -∆R1, -∆R2, -∆R3, -∆R4, -∆R5, -∆R6, -∆R7, and -∆C were constructed with an N-terminal flag tag by the mutagenesis of His-RCC2 plasmids using the Mutagenesis Kit (Vazyme, China), respectively. All of these constructs were confirmed by DNA sequencing.

All siRNAs were synthesized by GenePharma (Shanghai, China). A control siRNA (Qiagen, USA) that shows no homology to any known mammalian genes was used as a negative control. The sequences of the sense strands of the siRNA duplexes were as follows: 5ʹ-AAGAGATGAAAGTGAGACTGA-3ʹ (siRCC2-1), and 5ʹ-AAGGGGCAGCTGGGACATGGT-3ʹ (siRCC2-2).

### Cell culture, transfection, and drug treatment

HEK-293, HEK-293T, and HeLa cells were purchased from the Cell Bank of the Chinese Academy of Sciences (Shanghai, China) and cultured in Dulbecco’s modified Eagle’s Medium (Corning, USA) supplemented with 10% fetal bovine serum (FBS, ExCell Bio, China) at 37°C in 5% CO_2_. Plasmids were transfected with PolyJet (SignaGen Laboratories, Rockville, MD, USA), and the siRNA duplexes were transfected with Lipofectamine RNAiMAX (Invitrogen, Carlsbad, CA, USA). The transfection processes were performed according to the manufacturer’s instructions. For CHX chase analysis, 100 µg/mL CHX (Sigma-Aldrich, USA) was used as described in the text. HEK-293 cells were treated with 1 µmol/L MG132 (Millipore, Billerica, MA, USA) for 12 h to block the proteasome-dependent degradation pathway. Geldanamycin (GA, Tocris, UK) and radicicol (RA, Tocris, UK) were stored at −20°C as a stock solution of 5 mmol/L in DMSO and ethanol respectively. Cells were treated with geldanamycin for the indicated concentrations and times as described in the text.

### Generation of *NudCL2* KO cell lines by CRISPR/Cas9-mediated genome editing

The two sgRNAs, 5ʹ-GAAGTTCAGGTGCCGCCAGG-3ʹ and 5ʹ-TGGGATTCCGCGCGTGCGCTCGG-3ʹ, targeting the first exon of the NudCL2 gene were designed and synthesized by YSY Biotech Ltd (Nanjing, China). Then, the CRISPR/Cas9 plasmid was constructed by cloning the sgRNA into its backbone. HEK-293 and HeLa cells were transfected with this plasmid for 48 h followed by treatment with 1 μg/mL puromycin (Sigma-Aldrich, St. Louis, MO, USA) for 48 h. After selection, cells were counted and diluted to a density of 1 cell per 200 µL of medium and seeded into 96-well (Bio-Rad, Laboratories, Hercules, CA, USA) plates to obtain single colonies. *NudCL2* KO colonies were identified by Western blot and genomic DNA sequencing analyses. Two pairs of primers used to amplify the target regions are as follows:

*NudCL2* sgRNA-F1: 5ʹ-AGGCGTAGCCTAAGCGTGGGATTC-3ʹ;

*NudCL2* sgRNA-R1: 5ʹ-ACCCAACAGTCGTTCAGGGAAACG-3ʹ;

*NudCL2* sgRNA-F2: 5ʹ-ACTTAGGGGACGGTGTAGTGA-3ʹ;

*NudCL2* sgRNA-R2: 5ʹ-GGCGGCACCTGAACTTCAAT-3ʹ.

### Cell synchronization

Cell synchronization was performed as previously described (Capalbo et al., 2019; Compe et al., 2022). Briefly, HEK-293 cells were incubated with 2 mmol/L thymidine (Selleck, USA) for 18 h. After washing twice with phosphate-buffered saline (PBS), cells were released in fresh culture medium for 6 h. After release, cells were incubated with 2 mmol/L thymidine for another 18 h. Cells were released in fresh culture medium, harvested at the indicated time points by centrifugation at 1000 ×*g* for 3 min, frozen immediately in dry ice, and stored at −80°C. To synchronize cells in telophase, cells were synchronized with a double-thymidine block and released. Next, cells were cultured for an additional 13 h in fresh complete medium containing 20 ng/mL nocodazole (Sigma-Aldrich, USA) and then harvested by mitotic shake-off. Mitotic cells were washed three times with PBS, and released in fresh medium for 90 min to be collected in telophase.

### Antibodies

The following antibodies for Western blot (WB) and immunofluorescence (IF) were used in this study: two types of anti-NudCL2 antibodies were prepared, including a rabbit polyclonal anti-NudCL2 antibody that generated as described previously (Yang et al., 2010) and a mouse monoclonal antibody against NudCL2 peptide (GAEISGNYTKGGPDFSNLEK, 138–157 aa) as antigens. A rabbit polyclonal anti-RCC2 antibody was prepared (HuaBio, China) against the mixed peptides (RAGPRKRGGPAGRKRE, 22–37 aa and RVAIFIEKTKDGQILP, 311–326 aa) as antigens that were described in the previous study (Mollinari et al., 2003). The following antibodies and dilutions were commercially acquired for WB and IF analyses: mouse monoclonal anti-α-tubulin (Sigma-Aldrich, T6199 dilutions for WB 1:5,000, for IF 1:400), mouse monoclonal anti-β-actin (Sigma-Aldrich, T1978 dilution for WB 1: 5,000), mouse monoclonal anti-Myc (Cell Signaling Technology, 9B11 dilutions for WB 1:2,000, for IF 1:200), rabbit polyclonal anti-mAID (Novus Biologicals, NBP2-89163 dilutions for WB 1:1000), mouse monoclonal anti-His (Proteintech, 66005-1-Ig dilution for WB 1:1000), mouse monoclonal anti-GST (Santa Cruz Biotechnology, sc-138 dilution for WB 1:1000), rabbit polyclonal anti-Aurora B (Diagbio, db2045 dilutions for WB 1:2,000, for IF 1:200), rabbit polyclonal anti-MKLP1 (Diagbio, db5469 dilutions for WB 1:1000, for IF 1:100), rabbit polyclonal anti-PLK1 (Sigma-Aldrich, SAB4502211 dilutions for WB 1:2,000, for IF 1:200), rabbit polyclonal anti-MKLP2 (Proteintech, 15911-1-AP dilution for WB 1:1000), mouse monoclonal anti-ANXA2 (Proteintech, 60051-1-Ig dilution for WB 1:5,000), mouse monoclonal anti-GAPDH (ABclonal, AC002 dilution for WB 1:5,000), rabbit polyclonal anti-Hsp90 (Diagbio, db621 dilutions for WB 1:1000, for IF 1:100), and mouse monoclonal anti-Puromycin (Sigma-Aldrich, MABE343 dilutions for WB 1:10,000). The secondary antibodies used for immunofluorescence analysis were Alexa Fluor 488-and 568-conjugated anti-rabbit or anti-mouse IgG (Invitrogen). Goat anti-mouse or anti-rabbit secondary antibody (LI-COR, Lincoln, NE, USA) conjugated to either Alexa Fluor 680 or IRDye 800 was used for Western blot analysis.

### GST pull-down assay

GST pull-down assays were performed as described previously (Lu et al., 2017). In brief, GST, GST-NudCL2, His-RCC2, different types of His-tagged RCC2 truncates, including pET-28a-Flag-RCC2-∆R1, -∆R2, -∆R3, -∆R4, -∆R5, -∆R6, -∆R7 and-∆C- His, and His-Hsp90 proteins were purified from *Escherichia coli* BL21. The purified proteins were incubated in PBS at 4°C for 4 h, and then glutathione-agarose beads were added and incubated for 2 h. The beads were washed and then subjected to Western blot with the respective antibodies as indicated in the text.

### Immunoprecipitation and Western blot

Immunoprecipitation was performed as previously described (Yang et al., 2010). Briefly, cells were lysed in TBSN buffer [20 mmol/L Tris (pH 8.0), 150 mmol/L NaCl, 0.5% Nonidet P-40, 5 mmol/L EGTA, 1.5 mmol/L EDTA, 0.5 mmol/L Na_3_VO_4_, 20 mmol/L *p*-nitrophenyl phosphate] containing a cocktail of protease inhibitors (Roche, Basel, Switzerland) and then subjected to immunoprecipitation with the indicated antibodies. The proteins were separated in a sodium dodecyl sulfate (SDS)-polyacrylamide gel electrophoresis gel and transferred to a polyvinylidene fluoride membrane (Millipore, Billerica, MA, USA). The membranes were blocked with 5% BSA at room temperature for 1 h, incubated with the indicated primary antibodies followed by the secondary antibodies and then detected by ChemiDoc Touch Imaging System (Bio-Rad, USA) or LI-COR Odyssey imaging systems (LI-COR, USA).

### Immunofluorescence staining

Cells grown on microscope glass coverslips were fixed with 3.7% (*v*/*v*) paraformaldehyde at room temperature for 20 min or ice-cold methanol for 15 min at −20°C. After washing three times with PBST (0.1% Triton X-100 in PBS) for 15 min, cells were incubated in blocking buffer [3% (*w*/*v*) BSA in PBS] for 30 min at room temperature. The coverslips were incubated with the primary antibodies [diluted in PBS and 3% (*w*/*v*) BSA] indicated in the text for 2 h at room temperature. After washing with PBST for 15 min, cells were incubated with secondary antibodies (Alexa Fluor 488 or 568-conjugated anti-rabbit or mouse IgG, Invitrogen, USA) for 1 h at room temperature. DNA was stained with 4ʹ,6-diamidino-2-phenylindole (DAPI) (Beyotime Technology, Shanghai, China). The immunofluorescence pictures in Fig. S3 were captured by the highly intelligent and sensitive structured illumination microscopy (HIS-SIM, China) in 3D and reconstructed using Imaris software, while the other immunofluorescence pictures were captured in 2D by confocal fluorescence microscopy (Zeiss, LSM 800, Germany) and processed using ImageJ software.

### Time-lapse imaging

For time-lapse experiments, cells were plated on the μ-slides (4 well, Ibidi) and analyzed with differential interference contrast (DIC) images. DNA in live-cell experiment was stained for 15 min at 37°C with Hoechst 33342 NucBlue Live ReadyProbes Reagent (Invitrogen, Carlsbad, CA, USA). Imaging was performed on the OLYMPUS Spin10 (CSU-W1) inverted digital microscope. Images were collected with the UPLXAPO 40 × 1.40 NA OIL DIC 130 μm objective at 37°C in 5% CO_2_. We used the OLYMPUS cellSens Dimension 3.1 software for multidimensional image acquisition. Specimens were maintained at 37°C and 5% CO_2_ via a chamber, and z-series of sections were captured at 5 min intervals. All images were processed using ImageJ software to create the final movies.

### Proteomic analysis (iTRAQ) and quantification

Isobaric tags for the relative and absolute quantitation (iTRAQ) based proteomic analysis were performed as described previously (Lambert et al., 2013; Ross et al., 2004; Wisniewski et al., 2009). Briefly, samples including three independent repetitions of whole-cell lysates from WT, *NudCL2* KO-1, and *NudCL2* KO-2 cells were lysed in an SDT buffer [4% (*w*/*v*) SDS, 100 mmol/L Tris/HCI pH7.6, 0.1 mol/L DTT]. After centrifugation, the protein concentration of the supernatant was determined using BCA assay (Thermo Fisher Scientific, USA). An appropriate amount of protein from each sample was subjected to trypsin hydrolysis using the filter-aided protein preparation (FASP) method and then used C18 solid-phase extraction to desalt the peptides. Finally, the desalted peptide samples were dried in a vacuum concentrator for peptide iTRAQ labeling. The proteomic analysis of the samples was performed as iTRAQ 4-plex experiments according to the manufacturer’s instructions (AB-SCIEX, USA). The labeled peptides of each group were mixed and graded by Akta purifier 100. Then each graded sample was separated by HPLC liquid phase system. After chromatographic separation, the samples were analyzed by Q Exactive mass spectrometer (Thermo Scientific, USA). The original mass spectrometry file (raw) generated by Q Exactive mass spectrometer was analyzed with MASCOT2.2 (Matrix Science, London, UK; version 2.2) and Proteome Discoverer 1.4 (Thermo Fisher Scientific, USA) software for identification and quantitation analysis.

### RNA extraction and quantitative real-time PCR (qRT-PCR)

Total RNA was extracted with TRIzol (Invitrogen, USA) and reverse transcribed to obtain complementary DNA (cDNA) with HiScript II Q RT SuperMix (Vazyme, China). The LightCycler 480 II system (Roche) or CFX-96 (Bio-Rad) system was used to perform qRT-PCR using ChamQ Universal SYBR qPCR Master Mix (Vazyme, China). All of the reactions were performed in triplicate. Primers used to amplify the target region of *RCC2* mRNA are as follows: Forward: 5ʹ-GTGGGAAGAGCAGCATCATT-3ʹ; Reverse: 5ʹ-GAAGACTTGGGCTTGTGGTC-3ʹ.

### Midbody purification

Midbodies were purified according to the method previously reported (Capalbo et al., 2019). HEK-293 cells (at least 3 × 10^7^) were synchronized using the thymidine-nocodazole block and release procedure previously described. 5 μg/mL taxol (Selleck, USA) was added to the medium for 2–3 min to stabilize microtubules *in vivo* before collection. Then cells were collected by centrifugation at 250 ×*g* for 3 min. After washing once with pre-warmed H_2_O, cells were gently resuspended in 25 mL of swelling solution (1 mmol/L PIPES pH 7.0, 1 mmol/L MgCl_2_, 5 μg/mL taxol and Roche Complete Protease Inhibitors) and immediately centrifuged at 250 ×*g* for 3 min. The cell pellet was then resuspended in 40 mL of lysis buffer [1 mmol/L PIPES pH 7, 1% (*v*/*v*) NP-40, 1 mmol/L EGTA, 3 U/mL DNAse I, 10 μg/mL RNAse A, 1 U/mL micrococcal nuclease, 5 μg/mL taxol, and Roche Complete Protease Inhibitors] and vortexed vigorously for 1 min. After the addition of 0.3 volumes of cold 50 mmol/L 2-(N-morpholino) methanesulfonic acid (MES) pH 6.3, the sample was incubated on ice for 20 min and then centrifuged at 200 ×*g* for 10 min at 4°C, and the supernatant was transferred to a new tube and centrifuged at 650 ×*g* for 20 min at 4°C to pellet midbodies. The midbody pellet was then resuspended in 4 mL of 50 mmol/L MES pH 6.3 and centrifuged through a 25 mL glycerol cushion [40% (*w*/*v*) glycerol diluted in 50 mmol/L MES pH 6.3] at 2,800 ×*g* for 45 min at 4°C. After removal of the glycerol cushion, the midbody pellet was washed with 2 mL of 50 mmol/L MES pH 6.3, transferred to a 15 mL conical tube, and centrifuged at 2,800 ×*g* for 20 min at 4°C. The sample was then centrifuged at 3,500 ×*g* for 10 min at 4°C, the supernatant was carefully discarded and the pellet was left to dry for 5–10 min at room temperature. Precipitated proteins were stored at −80°C until further processing.

### Single-molecule fluorescent *in situ* hybridization (smFISH)

Single-molecule RNA FISH combined with immunofluorescence was performed as described previously (Querido et al., 2020; Raj et al., 2008; Tsanov et al., 2016). Briefly, mixed DNA probes for the detection of the transcripts of *RCC2* were designed and their sequences are listed in Appendix Table S1. Cells grown on coverslips were fixed with 4% PFA in PBS for 20 min and washed in PBS for three times (5 min each) and permeabilized in 75% ethanol overnight at 4°C. After rinsing once with PBS, the cells were washed with 10% formamide in 2× SSC for 10 min, followed by pre-hybridization in pre-hybridization buffer (10% formamide, 10% dextran sulfate in 2× SSC) at 37°C for 1 h. Subsequently, the cells were incubated with 488-labeled smFISH probes in hybridization buffer (10% dextran sulfate, 50% formamide, 1 mg/mL yeast tRNA, 5 mmol/L Ribonucleoside Vanadyl Complex in 1× SSC) for 18 h at 37°C (100 nmol/L probes). After three washes with 10% formamide in 2× SSC (5 min each) at 37°C, the follow-up immunofluorescence was carried out as described in the immunofluorescence staining section.

### Puromycin labeling assay

Puromycin labeling assay for the detection of nascent peptides was performed as described previously (Cook et al., 2014; Deliu et al., 2017). Briefly, cells were treated with 10 μg/mL puromycin (Sigma-Aldrich, P8833) for 30 min and fixed in 4% PFA in PBS for 20 min, then subjected to smFISH experiment to detect *RCC2* mRNA, followed by immunofluorescence with mouse anti-puromycin and rabbit anti-RCC2 antibodies. After three times washes with 0.1% Triton X-100 in PBS (5 min each), the cells were incubated with donkey anti-rabbit IgG conjugated with Alexa Fluor 555 and donkey anti-mouse IgG conjugated with Alexa Fluor 647 for 1 h.

For puromycin labeling of purified midbodies, cells were synchronized with a thymidine-nocodazole block/release, then treated with or without 10 μg/mL CHX for 30 min, and then incubated with 10 μg/mL puromycin for 30 min. Then the midbodies were purified as described in the midbody purification section and subjected to immunoprecipitation analysis using anti-RCC2 antibody.

### Auxin-inducible degron (AID) system

Auxin-inducible degron system was employed as described previously (Natsume et al., 2016; Nishimura et al., 2009). Briefly, lentiviruses were packaged with pLVX-OsTIR1-T2A-NudCL2-mAID or pLVX-OsTIR1-T2A-RCC2-mAID plasmids in HEK-293T cells, respectively, and the culture medium was harvested at 48 h. The *NudCL2* or *RCC2* KO HEK-293 cells were infected with the related lentiviruses-containing medium for 48 h, respectively, and followed by puromycin selection for another 48 h. Then, cells were synchronized in prometaphase using thymidine-nocodazole block and released for 30 min, then treated with phytohormone auxin indole3acetic acid to promote OsTIR1 binding to mAID and thereby induce mAID-NudCL2/RCC2 fusion protein rapid proteasomal degradation.

### Statistical analysis

All data presented are based on at least three biological replicates. Data are presented as the mean ±  SEM or SD. Two-tailed Student’s *t*-test was performed to determine statistically significant differences between the two groups, and comparisons between multiple groups were performed by one-way ANOVA. The statistical analyses of CHX chase experiments were performed using two-way ANOVA. Statistical analysis was performed using GraphPad Prism 8.4.0.

## Conclusion

We unveil the localization of NudCL2, Hsp90, and RCC2 at the midbody during cytokinesis, as well as the collaboration between NudCL2 and Hsp90 to stabilize RCC2 protein and ensure successful cytokinesis, which enhances our comprehension of the biological functions of the NudCL2 protein and provides fresh insights into the molecular mechanisms underlying cytokinesis.

## Supplementary data

The online version contains supplementary material available at https://doi.org/10.1093/procel/pwae025.

pwae025_suppl_Supplementary_Movie_S1

pwae025_suppl_Supplementary_Movie_S2

pwae025_suppl_Supplementary_Movie_S3

pwae025_suppl_Supplementary_Movie_S4

pwae025_suppl_Supplementary_Movie_S5

pwae025_suppl_Supplementary_Movie_S6

pwae025_suppl_Supplementary_Movie_S7

pwae025_suppl_Supplementary_Movie_S8

pwae025_suppl_Supplementary_Movie_S9

pwae025_suppl_Supplementary_Movie_S10

pwae025_suppl_Supplementary_Movie_S11

pwae025_suppl_Supplementary_Movie_S12

pwae025_suppl_Supplementary_Movie_S13

pwae025_suppl_Supplementary_Dataset_S1

pwae025_suppl_Supplementary_Dataset_S2

pwae025_suppl_Supplementary_Material

## Data Availability

All data needed to evaluate the conclusions in the paper are present in the paper and/or Supplementary Materials. The mass spectrometry proteomics data have been deposited at the ProteomeXchange Consortium via the PRIDE partner repository with the dataset identifier PXD043570.

## Code availability

All the custom codes are available from the corresponding authors upon reasonable request.
