# Supplementary material for: NudCL2 is required for cytokinesis by stabilizing RCC2 with Hsp90 at the midbody
Source: Protein Cell. 2024 May 27;15(10):766–82. doi: 10.1093/procel/pwae025 (PMC11443449; doi:10.1093/procel/pwae025)
Supplement: pwae025_suppl_Supplementary_Material [file pwae025_suppl_supplementary_material.pdf]

Supplementary information for

NudCL2 is required for cytokinesis by stabilizing RCC2 with Hsp90 at the midbody

Xiaoyang Xu[1,#], Yuliang Huang[1,#], Feng Yang[2], Xiaoxia Sun[1], Rijin Lin[1], Jiaxing Feng[1], Mingyang Yang[1], Jiaqi Shao[1], Xiaoqi Liu[3], Tianhua Zhou[1,4,5,*], Shanshan Xie[6,*] and Yuehong Yang[1, 4,*]

Corresponding authors:
yhyang@zju.edu.cn (Y. Yang);
sxie@zju.edu.cn (S. Xie);
tzhou@zju.edu.cn (T. Zhou)

This PDF file includes:

Figures S1 to S9
Table S1
Legends for Datasets S1 to S2

Other supporting materials for this manuscript include the following:

Movie S1-S13
Datasets S1 to S2

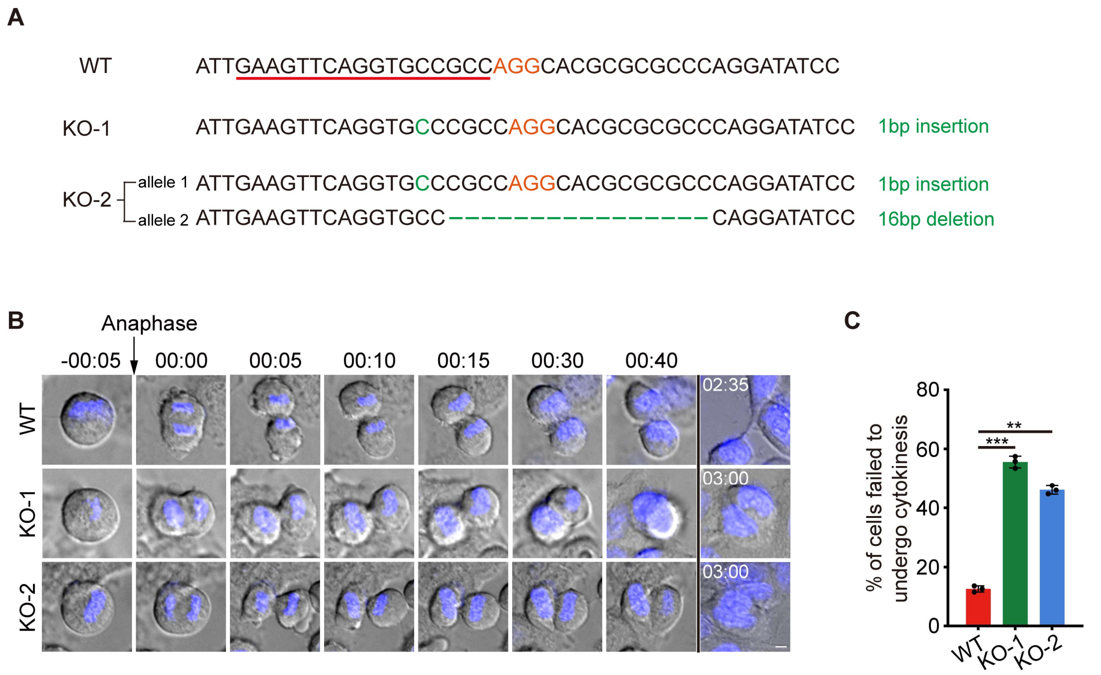

Figure S1 (related to Figure 1). Deletion of NudCL2 leads to cytokinesis failure

(A) Indel mutation of the *NudCL2* DNA locus in *NudCL2* knockout (KO) cells. The sgRNA target site (underlined) is indicated in red. The PAM (protospacer adjacent motif) site is indicated in orange. TA cloning of PCR products from genomic DNA extracted from WT and *NudCL2* KO cells. The green texts indicate base mutation (deletion or insertion) in the knockout cells. (B) Control and *NudCL2* KO HEK-293 cells were stained with the DNA-specific dye Hoechst 33342 for 15 minutes and subjected to time-lapse experiments. DIC stills of the live cell imaging experiment of the control or *NudCL2* KO cells. Time point 00:00 (hours:minutes) refers to the first frame where the separating sisters are observed. (C) Percentage of (n = 189), KO-1 (n = 157) or KO-2 (n = 155) cells showing cytokinesis failure was calculated, respectively. The P values were calculated using Student's t-test; $^{**}P < 0.01$, $^{***}P < 0.001$.

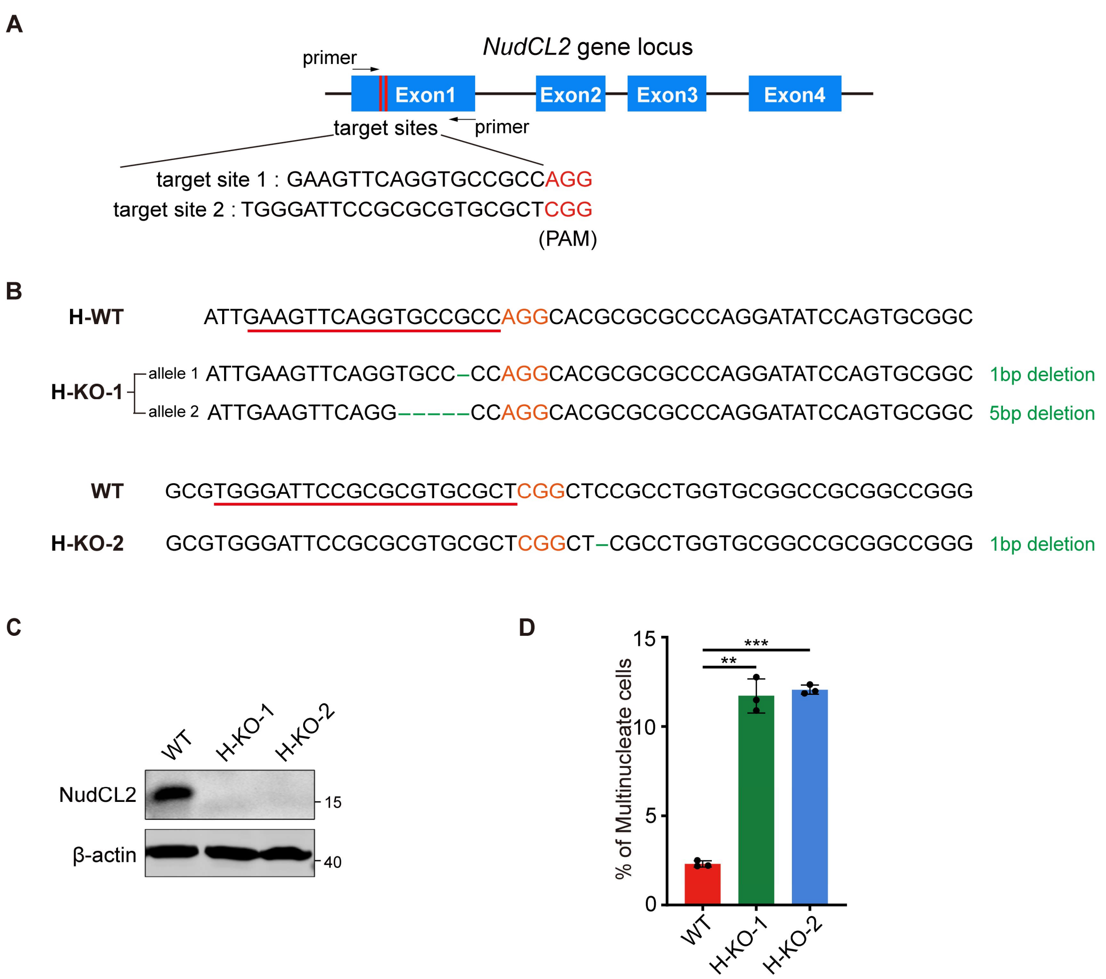

Figure S2 (related to Figure 1). Knockout of NudCL2 causes an increase in multinucleation in HeLa cells

(A) Schematic representation of *NudCL2* gene targeting strategy. (B) Indel mutation of the NudCL2 DNA locus in *NudCL2* KO HeLa cells. The sgRNA target sites (underlined) are indicated in red. The PAM sites are indicated in orange. TA cloning of PCR products from genomic DNA extracted from WT and *NudCL2* KO cells (H-KO-1 and H-KO-2). The green texts indicate base mutation (deletion) in the knockout cells. (C) Western blot analysis of NudCL2 protein in control and *NudCL2* KO cells. β-actin, a loading control. (D) Control and *NudCL2* KO cells were stained with DAPI and anti-α-tubulin antibody for immunofluorescence as described in Fig. 1J. The percentages of multinucleated cells in WT (n = 864), H-KO-1 (n = 638) and H-KO-2 (n = 588) were calculated. Quantitative data are expressed as the mean ± SD (from three biological replicates). Student's t-test; $^{**}P < 0.01$, $^{***}P < 0.001$,

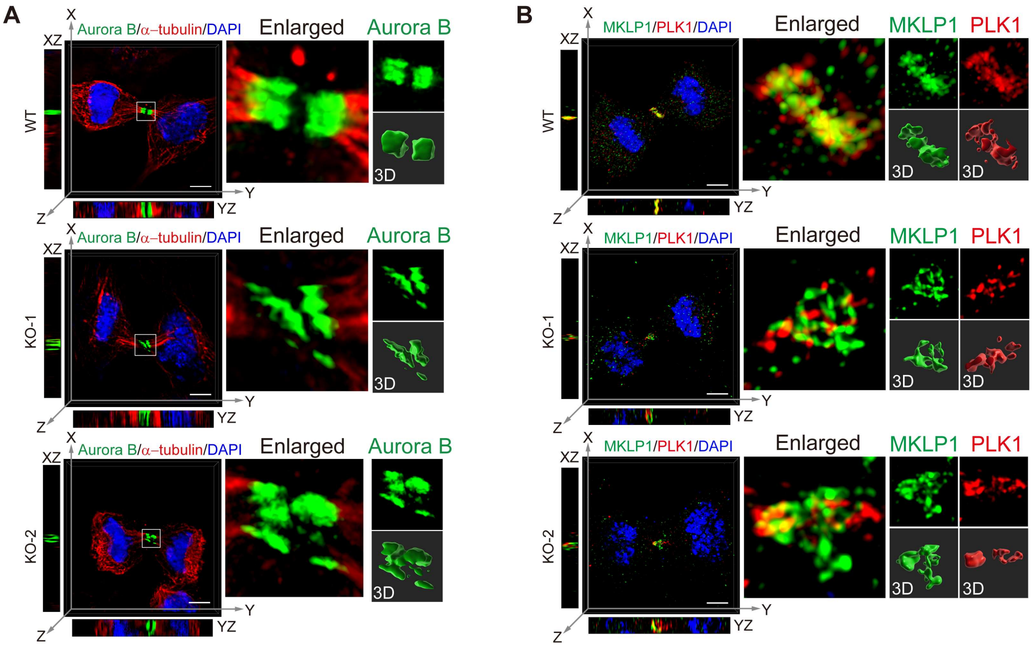

Figure S3 (related to Figure 2). Loss of NudCL2 disrupts the localization of Aurora B, PLK1 and MKLP1 at the midbody

(A and B) Control and *NudCL2* KO HEK-293 cells were fixed and subjected to immunofluorescence analysis with antibodies of Aurora B and α-tubulin (A) or MKLP1 and PLK1 (B). The super-resolution microscopy images and three-dimensional (3D) coordinate reconstructions showing the localization of Aurora B, PLK1 and MKLP1 at the midbody. Representative image with views at XZ and YZ planes. DNA was visualized with DAPI. Scale bars, 5 μm. Higher magnifications of the boxed regions are displayed.

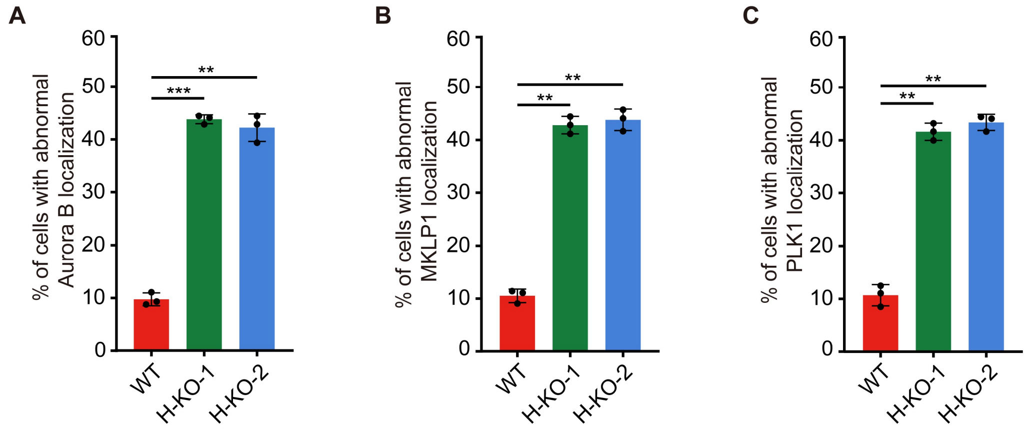

Figure S4 (related to Figure 2). Knockout of NudCL2 disrupts the midbody architecture in HeLa cells

(A) Control and *NudCL2* KO cells were fixed and stained with DAPI, anti-Aurora B and anti-α-tubulin antibodies for immunofluorescence as described in Fig. 2H. The frequencies of WT (n = 102), H-KO-1 (n = 105) and H-KO-2 (n = 104) cells with Aurora B mislocalization at the midbody were calculated. H-KO-1/ WT: *** P = 0.0001; H-KO-2/WT: ** P = 0.0026. (B, C) Control and *NudCL2* KO cells were fixed and stained with anti-MKLP1 and anti-PLK1 antibodies for immunofluorescence as described in Fig. 2J. The frequencies of cells with mislocalization of MKLP1 (n = 104, 105, 105) or PLK1 (n = 103, 108, 106) at the midbody were calculated. Quantitative data are expressed as the mean ± SD (from three biological replicates). Student's t-test; **$P < 0.01$, ***$P < 0.001$.

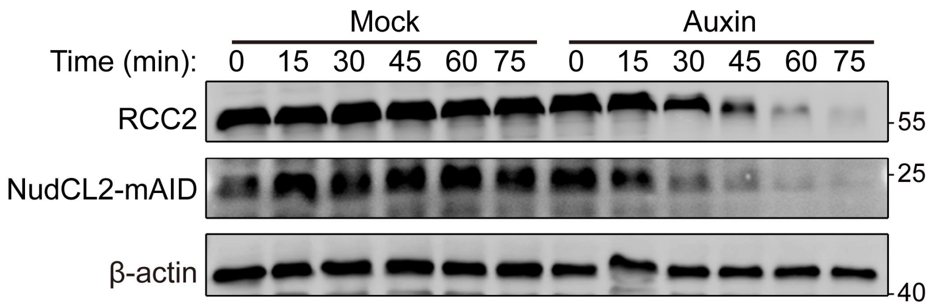

Figure S5 (related to Figure 3). Rapid downregulation of NudCL2 protein by mAID system induces the decrease of RCC2 protein.

The NudCL2 KO HEK-293 cells expressing OsTIR1-T2A-NudCL2-mAID were synchronized into anaphase by thymidine-nocodazole blocking and releasing for 30 minutes. Then cells were treated with or without auxin and harvested immediately at different time points, and subjected to western blot using the indicated antibodies. β-actin, a loading control.

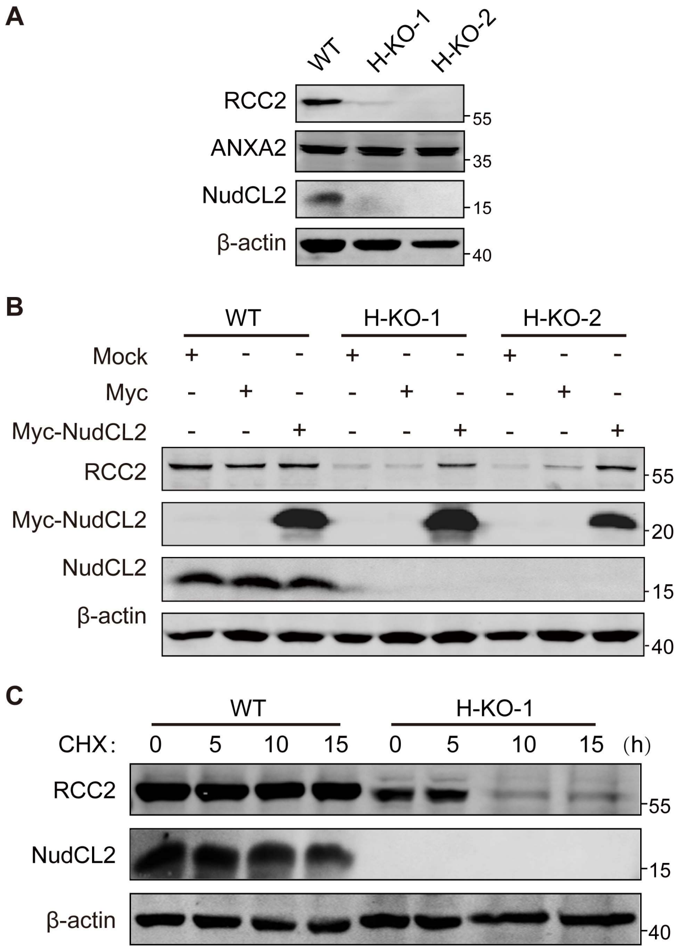

Figure S6 (related to Figure 3). Knockout of NudCL2 decreases the stability of RCC2 protein in HeLa cells

(A) Western blot analysis of protein extracts from control and *NudCL2* KO cells using the indicated antibodies. (B) Western blot analysis of protein extracts from control and *NudCL2* KO cells transfected with or without Myc or Myc-NudCL2 vector with the indicated antibodies. (C) Control and *NudCL2* KO-1 cells were treated with 100 μg/ml CHX and subjected to western blot analysis using anti-RCC2 and anti-NudCL2 antibodies. β-actin, a loading control.

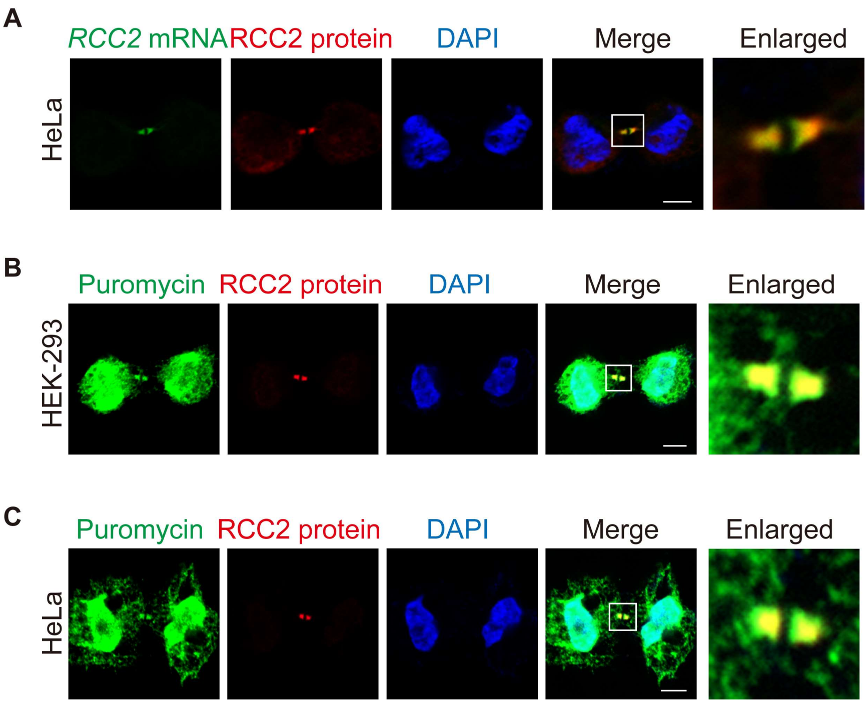

Figure S7 (related to Figure 3). The protein of RCC2 co-localizes with its mRNA and the puromycin signals at the midbody.

(A) HeLa cells were fixed and subjected to single molecule RNA fluorescence in situ hybridization (smFISH) to detect *RCC2* mRNA, then followed by immunofluorescence analysis with anti-RCC2 antibody. (B and C) Cells labeled with puromycin for 30 min were fixed and subjected to immunofluorescence analysis with the antibodies as shown. DNA was visualized with DAPI. Scale bars, 5 μm. Higher magnifications of the boxed regions are displayed.

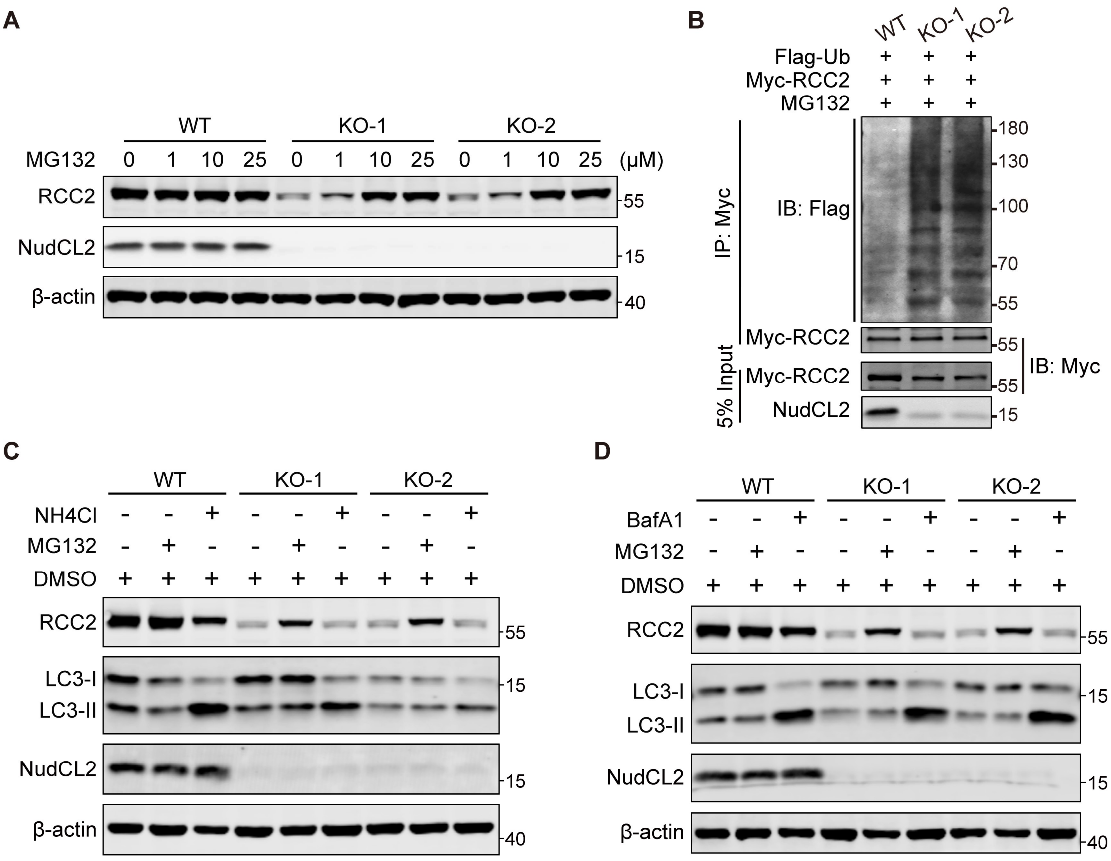

Figure S8 (related to Figure 3). RCC2 protein may be mainly degraded by the ubiquitin-dependent proteasome pathway in *NudCL2* KO cells.

(A) Control and *NudCL2* KO HEK-293 cells were treated with 1, 10 or 25 μM MG132 for 6 h and subjected to western blot with the indicated antibodies. (B) WT or NudCL2 KO HEK-293 cells were transfected with Myc-RCC2 and Flag-Ub for 48 h, then treated with 10 μM MG132 for 6 h and subjected to immunoprecipitation with anti-Myc antibody followed by western blot analysis with the indicated antibodies. 5% of total input is shown. (C and D) Control and *NudCL2* KO HEK-293 cells were treated with 10 μM MG132, 20 mM ammonium chloride (NH_4Cl) (C), and 100 nM bafilomycin A1 (BafA1) (D) for 2 h, followed by western blot with the indicated antibodies. β-actin, a loading control.

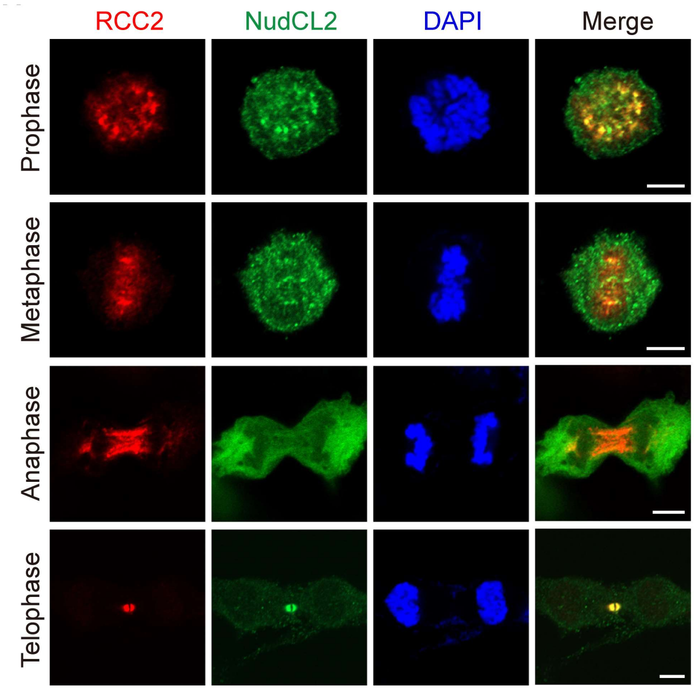

Figure S9 (related to Figure 3). The localization of RCC2 and NudCL2 during mitosis.

HEK-293 cells were fixed and subjected to immunofluorescence analysis with the indicated antibodies. DNA was visualized with DAPI. Scale bars, 5 μm.

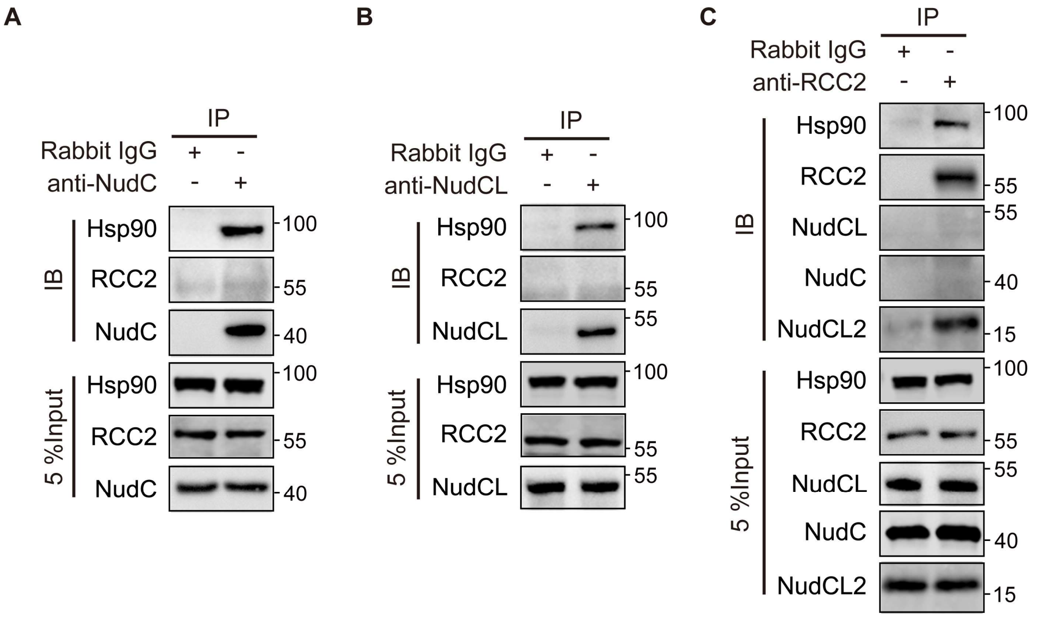

Figure S10 (related to Figure 4). NudCL2 specifically interacts with Hsp90 and RCC2.

(A-C) Immunoprecipitation experiments were performed using anti-NudC (A), anti-NudCL (B), or anti-RCC2 (C) antibodies, respectively, then followed by western blot analyses using antibodies as shown. 5% of total input is shown.

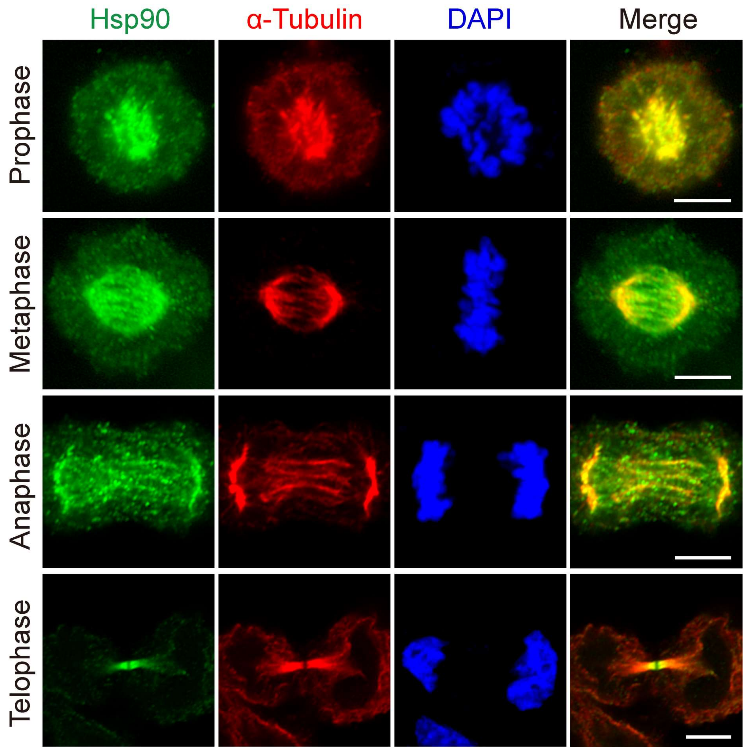

Figure S11 (related to Figure 5). The localization of Hsp90 during mitosis. HEK-293 cells were fixed and subjected to immunofluorescence analysis with the indicated antibodies. DNA was visualized with DAPI. Scale bars, 5 μm.

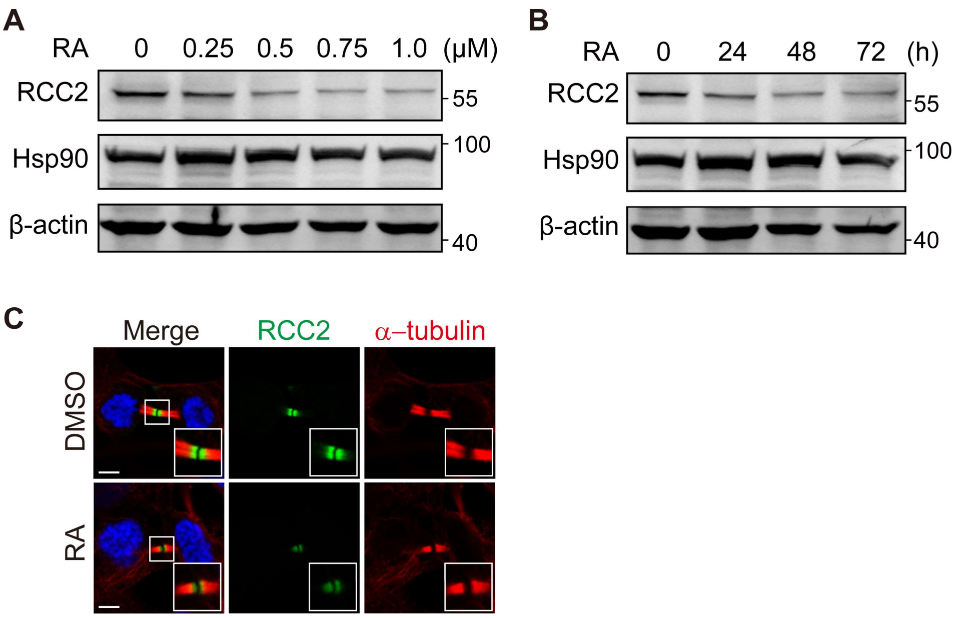

Figure S12 (related to Figure 5). Inhibition of Hsp90 ATPase activity by RA decreases the RCC2 protein at the midbody

(A and B) HEK-293 cells treated with different concentrations of RA for 48 h (A) or 0.5 μM RA at the indicated time points (B) were subjected to western blot analyses with the indicated antibodies. β-actin, a loading control. (C) Cells treated with 0.5 μM RA for 48 h were subjected to immunofluorescence analysis with the indicated antibodies. Scale bars, 5 μm.

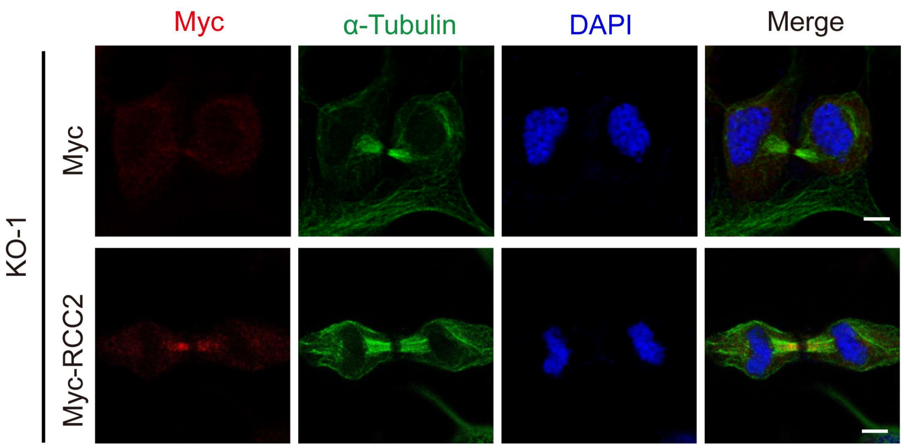

Figure S13 (related to Figure 6). Ectopically expressed Myc-RCC2 is able to localize at the midbody in *NudCL2* KO cells

The *NudCL2* KO HEK-293 cells were transfected with Myc or Myc-RCC2 for 48 h and subjected to immunofluorescence analysis with anti-Myc and anti-α-tubulin antibodies. DNA was visualized with DAPI. Scale bars, 5 μm.

Table S1 (related to Figure 3). List of the smFISH probes for the detection of *RCC2* mRNA.

Number	Name	Sequence 5'-3'
1	RCC2-P1	gccgccgccgccttcttcct
2	RCC2-P2	gttgcccgagctcggctcct
3	RCC2-P3	agctccaggccgtcctcgtc
4	RCC2-P4	ctcgggttcggtgatgacca
5	RCC2-P5	cttcaagtttgacgcgctcc
6	RCC2-P6	aagctgccctttgcactttg
7	RCC2-P7	tcgaccaatcaagtcccagt
8	RCC2-P8	cggtaagcagcttgctgttt
9	RCC2-P9	gccaggcacccatatctgtg
10	RCC2-P10	agcacacgagcccgagacca
11	RCC2-P11	tcatttcgaccccagctcca
12	RCC2-P12	cgcccacatgctgcagacac
13	RCC2-P13	gagcccgtttccgtcaaggc
14	RCC2-P14	agctgccccatcttgttttc
15	RCC2-P15	tgtacattatctgcgcgggg
16	RCC2-P16	attatcatactgaattcagc
17	RCC2-P17	aatagaggtttcctttgcag
18	RCC2-P18	gagttgtgtcccagctgacc
19	RCC2-P19	tactctatccgctgtgcccg
20	RCC2-P20	atgaagatggccactcgccg
21	RCC2-P21	acaggcagaatctgtccatc
22	RCC2-P22	gccacgtctcgtacaaccac
23	RCC2-P23	ccaggaccagcgtgtggtta
24	RCC2-P24	cggccatagccaccaaagcc
25	RCC2-P25	atctcatccttctgctctgc
26	RCC2-P26	gtcaaacagcttcaccaggc

27	RCC2-P27	caggtgtaaccagcatagat
28	RCC2-P28	aacagaccacccacttcact
29	RCC2-P29	cgcagaggtcctgcactgct
30	RCC2-P30	gctgctcttcccacaagcca
31	RCC2-P31	cagctgatggtgctctcatc
32	RCC2-P32	ccccgtagcccagttcccca
33	RCC2-P33	tccagagtctttacctcctg
34	RCC2-P34	ccatggcgacctgctctgag
35	RCC2-P35	tcatctcttgctatcaccaa
36	RCC2-P36	ggttcgggggttgtattctg
37	RCC2-P37	tgcacatggaaatgacagct
38	RCC2-P38	aaattcctcgtttgacttcc
39	RCC2-P39	cggaacctcagggagtcta
40	RCC2-P40	ggtagttaacgttggatcat
41	RCC2-P41	ggactttggaaagcatacag
42	RCC2-P42	aaatcaactatgagcaagta
43	RCC2-P43	acctagattgctcaaagttt
44	RCC2-P44	acgatgctaattgtaactgg
45	RCC2-P45	ctcttccatgtggcatctgc

Dataset S1 (separate file related to Figure 3). Quantitative proteomic analysis in WT, *NudCL2* KO-1 and *NudCL2* KO-2 HEK-293 cells

Quantitative proteomic analysis based on the isobaric tags for relative and absolute quantitation (iTRAQ) labeling in WT, *NudCL2* KO-1 and *NudCL2* KO-2 HEK-293 cells.

Dataset S2 (separate file related to Figure 3). List of the midbody proteins of human selected from MiCroKiTS 4.0 database

The list of the midbody proteins of human selected from MiCroKiTS 4.0 database (http://microkit.biocuckoo.org/).
